# Plant-Derived Caffeic Acid and Its Derivatives: An Overview of Their NMR Data and Biosynthetic Pathways

**DOI:** 10.3390/molecules29071625

**Published:** 2024-04-04

**Authors:** Jiahui Yu, Jingchen Xie, Miao Sun, Suhui Xiong, Chunfang Xu, Zhimin Zhang, Minjie Li, Chun Li, Limei Lin

**Affiliations:** 1 Key Laboratory for Quality Evaluation of Bulk Herbs of Human Province, School of Pharmacy, Human University of Chinese Medicine, Changsha 410208, China; 20223725@stu.hnucm.edu.cn (J.Y.); xiejingchen2022@163.com (J.X.); 20223726@stu.hnucm.edu.cn (M.S.); 20222058@stu.hnucm.edu.cn (S.X.); xcf200002@163.com (C.X.); cslgdxzzm@163.com (Z.Z.); tfxiaobei@163.com (M.L.); 2 Institute of Chinese Materia Medica, China Academy of Chinese Medical Sciences, Beijing 100700, China; cli@icmm.ac.cn

**Keywords:** caffeic acid, caffeic acid derivatives, NMR, biosynthetic pathway

## Abstract

In recent years, caffeic acid and its derivatives have received increasing attention due to their obvious physiological activities and wide distribution in nature. In this paper, to clarify the status of research on plant-derived caffeic acid and its derivatives, nuclear magnetic resonance spectroscopy data and possible biosynthetic pathways of these compounds were collected from scientific databases (SciFinder, PubMed and China Knowledge). According to different types of substituents, 17 caffeic acid and its derivatives can be divided into the following classes: caffeoyl ester derivatives, caffeyltartaric acid, caffeic acid amide derivatives, caffeoyl shikimic acid, caffeoyl quinic acid, caffeoyl danshens and caffeoyl glycoside. Generalization of their ^13^C-NMR and ^1^H-NMR data revealed that acylation with caffeic acid to form esters involves acylation shifts, which increase the chemical shift values of the corresponding carbons and decrease the chemical shift values of the corresponding carbons of caffeoyl. Once the hydroxyl group is ester, the hydrogen signal connected to the same carbon shifts to the low field (1.1~1.6). The biosynthetic pathways were summarized, and it was found that caffeic acid and its derivatives are first synthesized in plants through the shikimic acid pathway, in which phenylalanine is deaminated to cinnamic acid and then transformed into caffeic acid and its derivatives. The purpose of this review is to provide a reference for further research on the rapid structural identification and biofabrication of caffeic acid and its derivatives.

## 1. Introduction

Caffeic acid (CA), also known as 3,4-dihydroxy cinnamic acid, is an organic compound that has two functional groups (phenolic hydroxyl and acrylic acid) [1]. Caffeic acid derivatives refer to a large class of compounds that contain caffeic acid structural units [2]. Caffeic acid and its derivatives are widely distributed in medicinal plants, vegetables and fruits [3]. As a kind of safe and effective natural phenolic acid compound with a wide range of sources, caffeic acid exhibits many pharmacological effects, such as antioxidation [4], antibacterial [5], antiviral [6], antitumor [7], anti-inflammatory [8] and neuroprotection [9] effects and the ability to regulate blood glucose and blood lipids [10].

This paper summarizes the structural and Nuclear Magnetic Resonance Spectroscopy (NMR) spectral features of plant-derived caffeic acid and its derivatives due to their physiological activities and wide distribution in nature. The results provide a reference for the rapid structural identification of these compounds. The process of extracting these compounds from plants is complicated and affected by the plant growth cycle, climatic environment and other factors; thus, the plant cannot provide stable raw materials for natural product extraction, which greatly limits its large-scale production. Therefore, the biosynthetic pathways that generate caffeic acid and its derivatives are summarized and found to mainly involve the shikimic acid pathway, from which phenylalanine is deaminated to cinnamic acid and then converted into caffeic acid [11].

**Figure 1:**
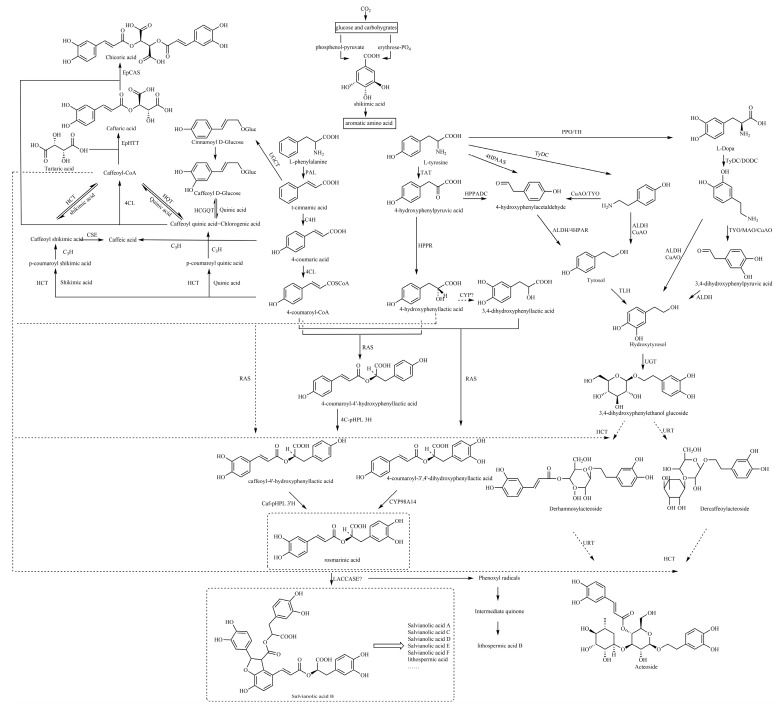
The biosynthesis pathway of caffeic acid and its derivatives. PAL, phenylalanine ammonia-lyase; C4H, cinnamic acid 4-hydroxylase; 4CL, 4-coumarate CoA ligase; TAT, tyrosine aminotransferase; HPPR, 4-hydroxyphenylpyruvate reductase; RAS, rosmarinic acid synthase, hydroxycinnamoyl-CoA: hydroxyphenyllactate hydroxycinnamoyltransferase; 3-H and 3′-H, 4C-pHPL 4-coumaroyl-4′-hydroxyphenyllactate 3/3′-hydroxylases; 3-H and 3′-H, Caf-pHPL caffeoyl-4′-hydroxyphenyllactate 3/3′-hydroxylase; CYP98A14, cytochrome P450-dependent monooxygenase; UGCT, UDP glucose: cinnamate glucosyl transferase; HCGQT, hydroxycinnamoyl D-glucose: quinate hydroxycinnamoyl transferase; HQT, hydroxycinnamoyl CoA quinate hydroxycinnamoyl transferase; C3H, p-coumarate 3-hydroxylase; HCT, hydroxycinnamoyl CoA shikimate hydroxycinnamoyl transferase; CSE, caffeoyl shikimate esterase; PPO, polyphenol oxidase; TH, tyrosine hydroxylase or tyrosine3-monooxygenase; TyDC, tyrosine or L-dihydroxyphenylalanine (DOPA) decarboxylase; 4HPAAS, 4-hydroxyphenylacetaldehyde synthase; HPPADC, 4-hydroxyphenylpyruvate decarboxylase; TYO, tyramine oxidase; CuAO, copper amine oxidase; DODC, Amino acid decarboxylase or dopa decarboxylase; MAO, monoamine oxidase; ALDH, alcohol dehydrogenase or arylalchohol dehydrogenase; 4HPAR, 4-hydroxyphenylpyruvate reductase; TLH, tyrosol hydroxylase; UGT, UDP-glucose: glycosyltransferase; URT, UDP-rhamnose glucosyltransferase; EpCAS, chicoric acid synthase; EpHTT, tartaric acid hydroxycinnamoyl transferase.

**Figure 2:**
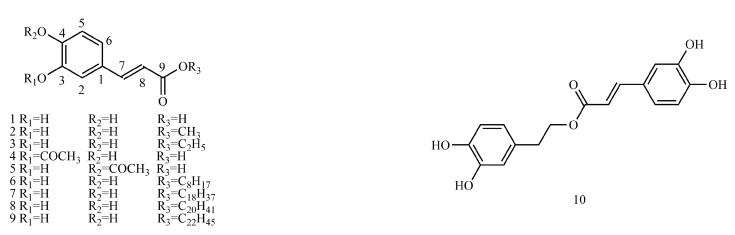
Structures of caffeic acid and caffeoyl ester derivatives.

**Figure 3:**
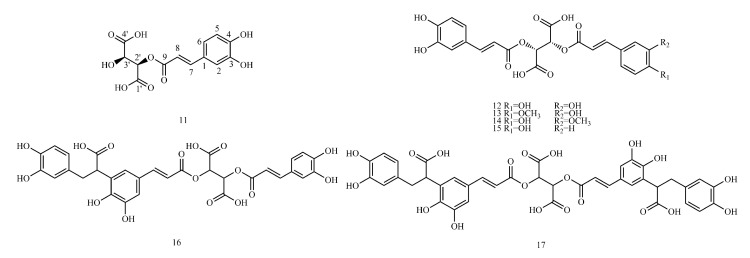
Structures of caffeyltartaric acids.

**Figure 4:**
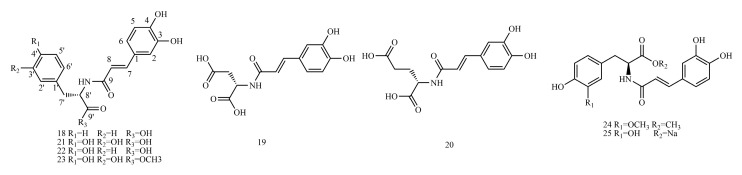
Structures of caffeic acid amide derivatives.

**Figure 5:**
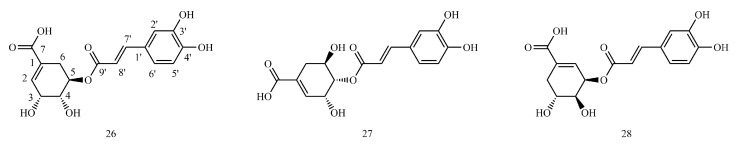
Structures of caffeoyl shikimic acids.

**Figure 6:**
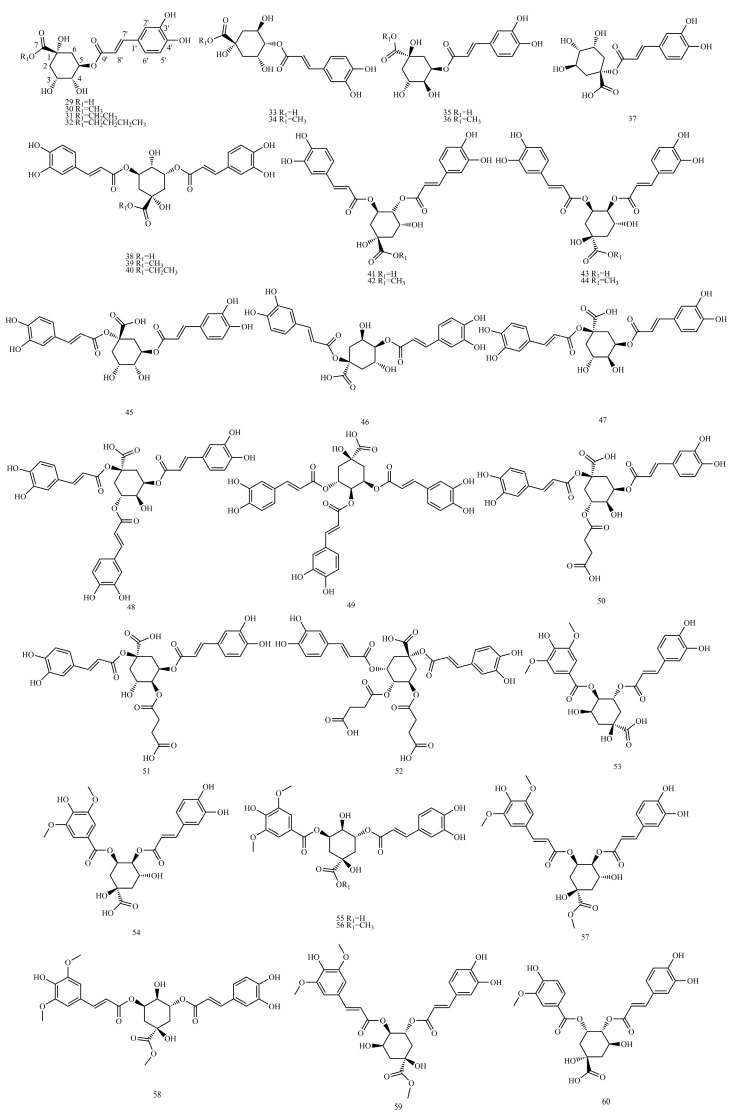

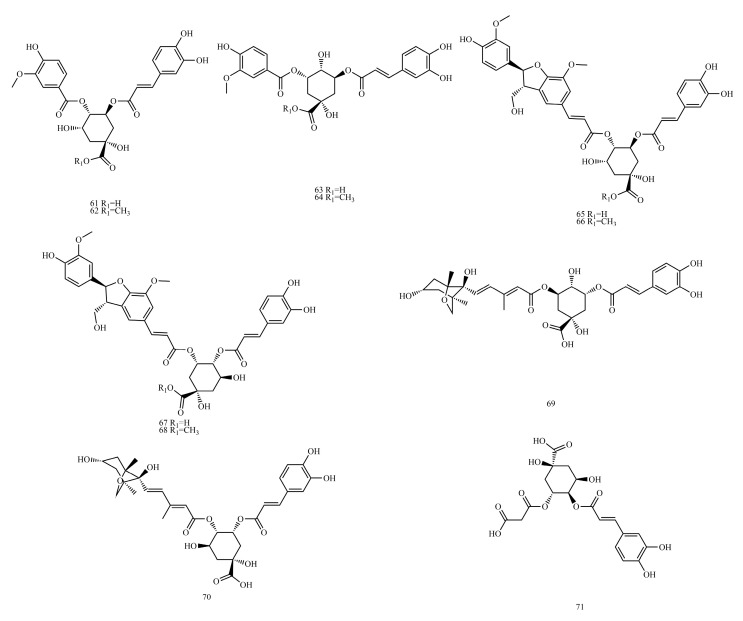
Structures of caffeoyl quinic acids.

**Figure 7:**
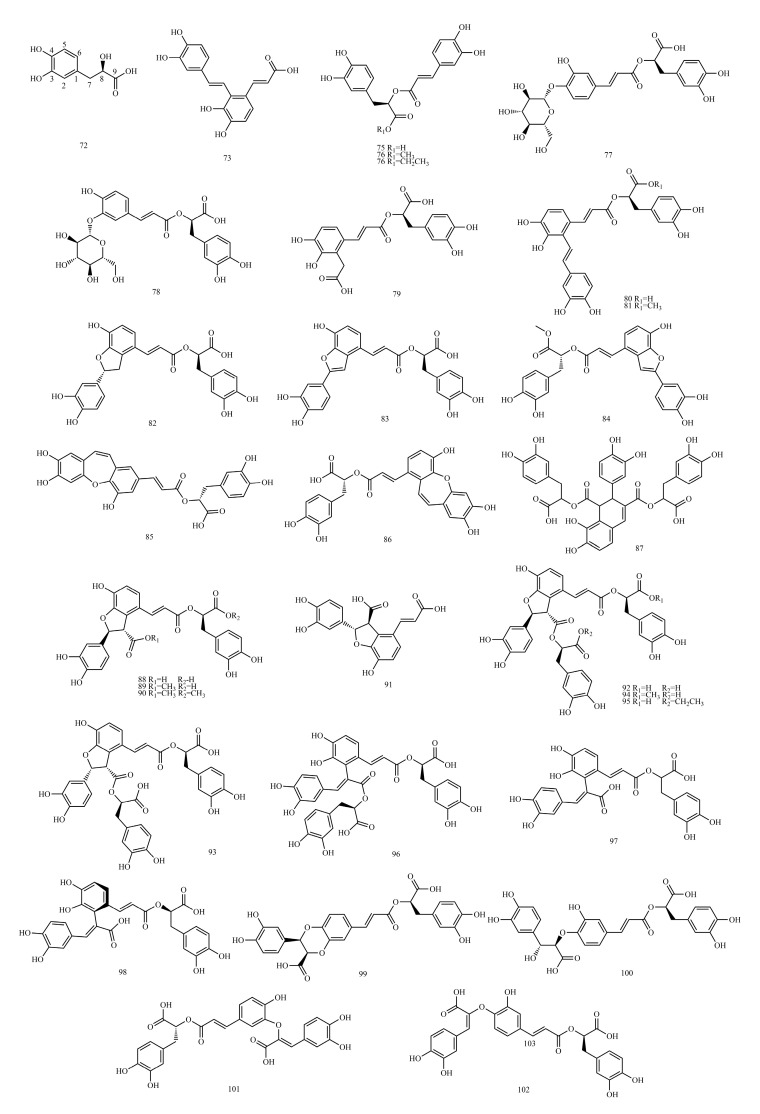
Structures of caffeoyl danshensu.

**Figure 8:**
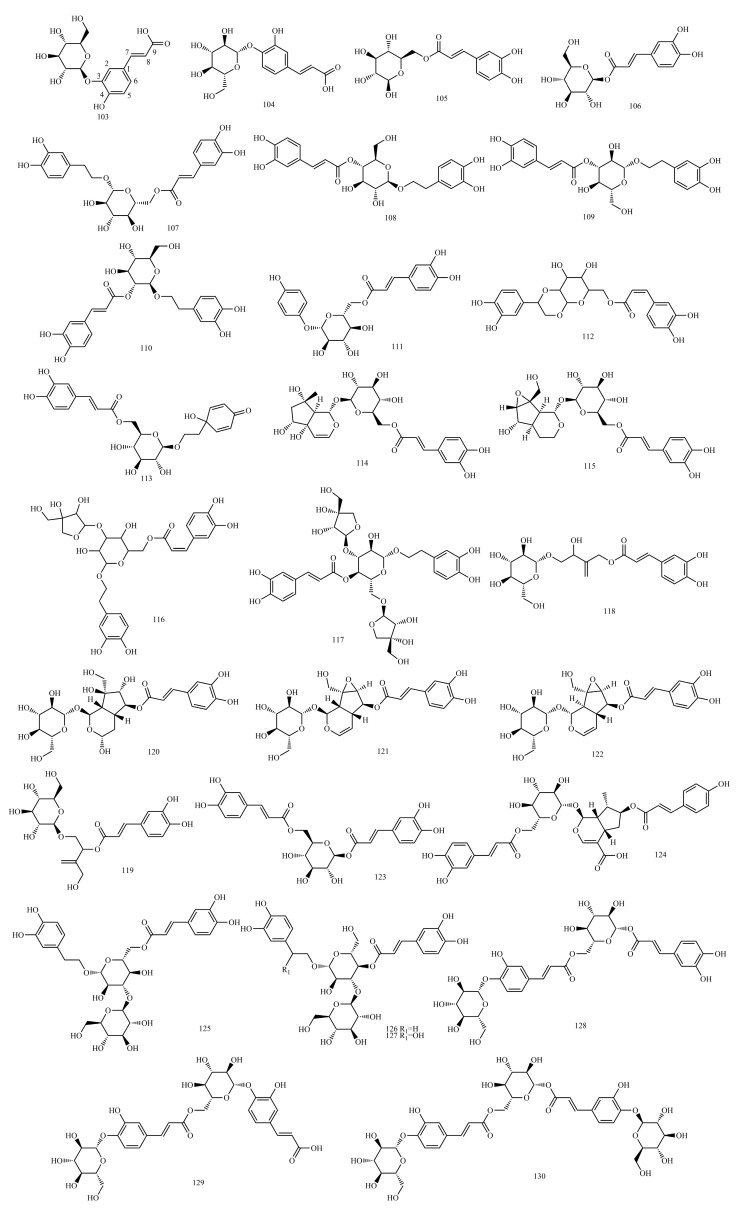

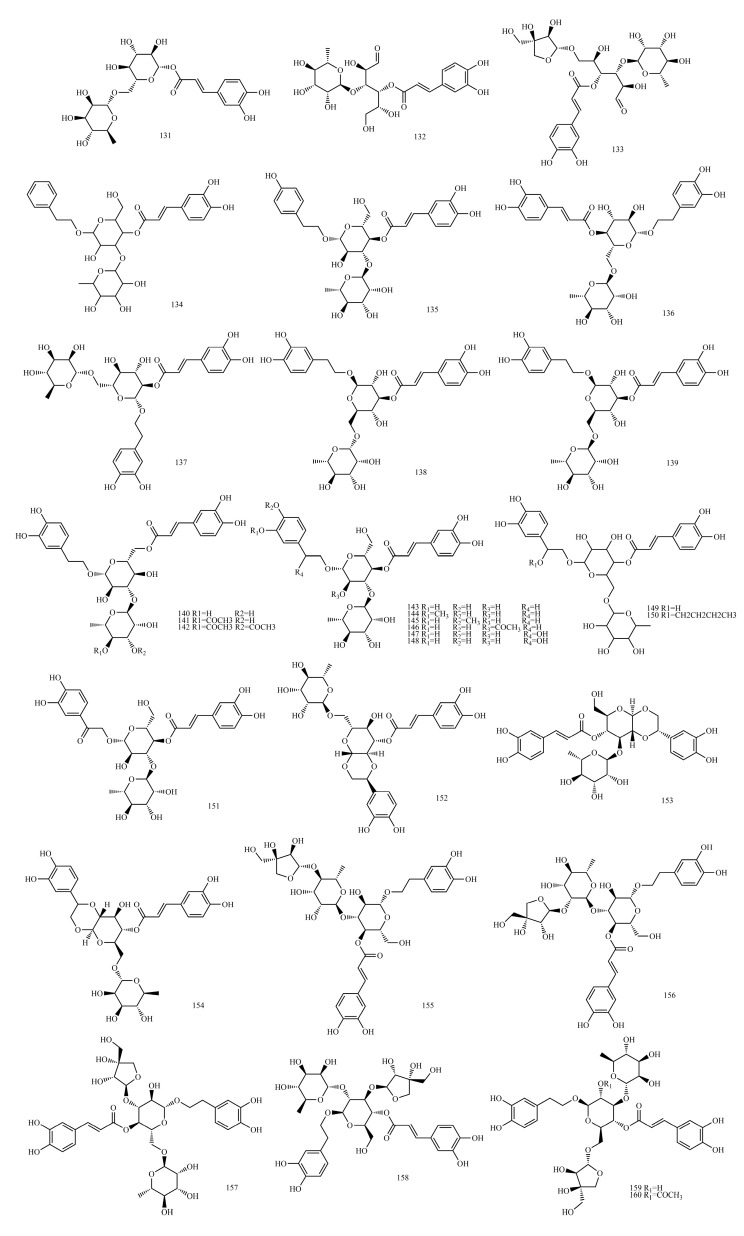

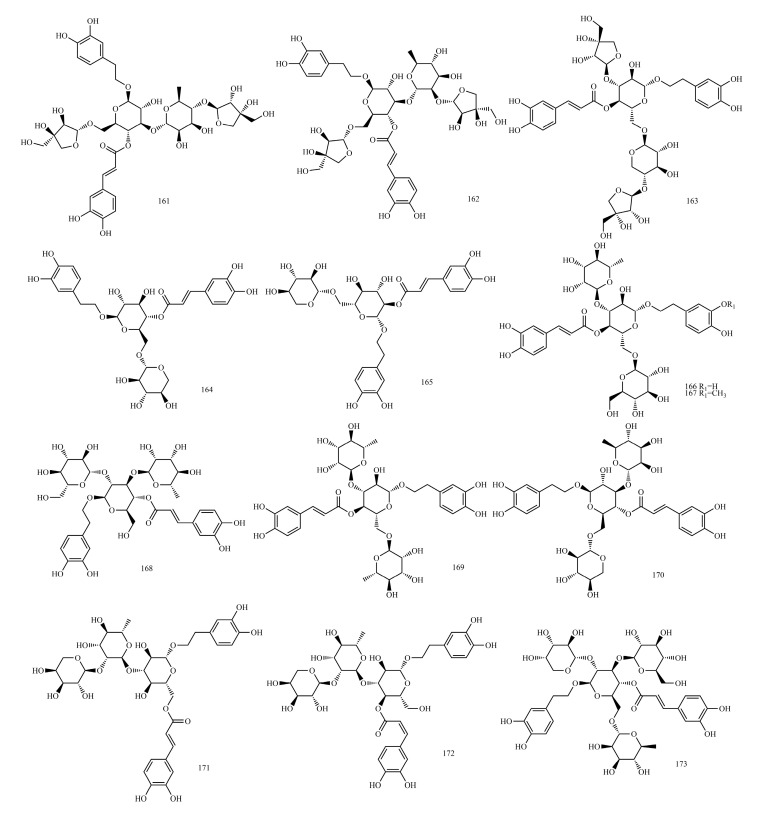
Structures of caffeoyl glycosides.

Therefore, ^13^C-NMR and ^1^H-NMR data (Tables S1–S18) and biosynthetic pathways (Figure 1) of 173 caffeic acid and its derivatives on plants with different types of substituents (Figure 2, Figure 3, Figure 4, Figure 5, Figure 6, Figure 7 and Figure 8 and Table 1, Table 2, Table 3, Table 4, Table 5, Table 6 and Table 7) were summarized to provide a reference for further research on the structural identification and biofabrication of caffeic acid and its derivatives.

## 2. Methodology

A comprehensive survey of the structural information, NMR data and biosynthetic pathways of caffeic acid and its derivatives was conducted by searching the scientific literature published in online databases (including PubMed, CNKI and SciFinder) and other sources (such as Ph. D. dissertations and M. Sc. theses). The search terms “caffeic acid”, “caffeic acid derivatives”, “caffeic acid and NMR”, “caffeic acid derivatives and NMR”, “caffeic acid and biosynthetic pathways” and “caffeic acid derivatives and biosynthetic pathways” were used for data collection. In total, 162 publications were included from 1984 to 2023. EndNote was used to collate published literature. To classify caffeic acid derivatives according to their structures, ChemDraw 20.0 software was used to draw chemical structures.

## 3. Structure and Classification of Caffeic Acid and Its Derivatives

In this paper, 1743 caffeic acid and its derivatives are compared. The skeletons of these caffeic acid derivatives can be classified into the following types according to the type of substituent: caffeoyl ester derivatives (Figure 1 and Table 1), caffeyltartaric acid (Figure 2 and Table 2), caffeic acid amide derivatives (Figure 3 and Table 3), caffeoyl shikimic acid (Figure 4 and Table 4), caffeoyl quinic acid (Figure 5 and Table 5), caffeoyl danshensu (Figure 6 and Table 6) and caffeoyl glycoside (Figure 7 and Table 7).

Caffeoyl ester derivatives are mainly synthesized by the ester formation of caffeic acid with different alcohols. Caffeic acid amide derivatives are produced by the condensation reaction between caffeic acid and amino acids. Caffeoyl tartaric acids are produced by the condensation of tartaric acid and caffeic acid through esterification. Caffeoyl shikimic acid is condensed from shikimic acid and caffeic acid by an esterification reaction. Caffeoyl quinic acid is a class of phenolic acid natural ingredients formed by the condensation of quinic acid with a varying number of caffeic acids through esterification. Because the carboxyl group of caffeic acid and the three hydroxyl groups on the alicyclic ring of quinic acid mangiferylate are easily acylated, the isomers are particularly abundant. Caffeoyl danshensu is formed by the esterification and condensation of caffeic acid and its hydrated product 3,4-dihydroxyphenyllactic acid. The main types of sugars in caffeoyl glycoside are glucose, rhamnose, xylose, furanose and glucuronic acid. The classification of sugar type mainly depends on acid hydrolysis, gas chromatography-mass spectrometry (GC–MS), NMR and other technologies.

## 4. ^13^C-NMR and ^1^H-NMR Data of Caffeic Acid and Its Derivatives

First, the number of caffeoyl groups was determined by ^13^C-NMR and ^1^H-NMR, and there were five hydrogen proton signals in the ^1^H-NMR (CD_3_OD, 500 MHz) of caffeic acid. In the aromatic region, *δ*_H_ 6.99 (1H, d, *J* = 1.8 Hz), 6.84 (1H, dd, *J* = 1.8, 8.2 Hz) and 6.73 (1H, d, *J* = 8.2 Hz) are characteristic signals for hydrogen protons of the benzene-ring ABX system. *δ*_H_ 7.27 (1H, d, *J* = 15.9 Hz) and 6.28 (1H, d, *J* = 15.9 Hz) are the characteristic signals for the hydrogen of adjacent alkenes in trans-double bonds. The chemical shifts of the double-bonded α and β hydrogens on the side chain are strongly influenced by the terminal carbonyl conjugation effect, with the α-H located in the higher field (*δ*_H_ 6.2~6.5) and the β-H located in the lower field (*δ*_H_ 7.4~7.7). There were nine carbon signals in the ^13^C-NMR spectrum (CD_3_OD, 125 MHz), of which *δ*_C_ 147.9, 146.5, 129.3, 123.1, 121.7 and 116.4 were carbon signals of the benzene ring skeleton. *δ*_C_ 141.5 and 114.6 were trans-double-bonded carbon signals, and *δ*_C_ 176.2 was a carboxyl carbon signal [128]. The cis-alkenyl carbon is more abundant than the trans-alkenyl carbon. The side chain α and β double bond structures can be used to determine the cis-trans isomers by the coupling constants of the alkene protons. In the ^1^H-NMR, most compounds have trans-alkene bonding signals *δ*_H_ 7.61, 6.35 (each 1H, *J* = 16.0 Hz, -CH=CH-), and a few have cis-alkene bonding signals *δ*_H_ 5.93 (1H, d, *J* = 12.8 Hz) and 7.11 (1H, d, *J* = 12.8 Hz). The α and β double bonds are not as structurally stable as in the trans form if they are cis-substituted [129].

The structure of monacyl compounds can be determined by 1D NMR spectroscopy. For disubstituted or more substituted compounds, a 2D NMR spectrum is needed to accurately localize the linkages. First, the parent nucleus is determined, and then the substituent position is determined. Generally, the hydrogen on the 3, 4hydroxyl groups and 9 carboxy groups of caffeic acid is replaced.

### 4.1. Caffeoyl Ester Derivatives

Table S1 shows the ^13^C-NMR and ^1^H-NMR data of caffeoyl ester derivatives.

Examples are as follows. See Table 8 below.

#### 4.1.1. ^1^H-NMR Data Obtained for Caffeoyl Ester Derivatives

When C_3_-OH, C_4_-OH or C_9_-OH are esterified, there is little effect on the chemical shift values of H-2, H-5 or H-8. As the induced effect is transmitted through bonding electrons, the influence of the induced effect diminishes with increasing distance from the electronegative substituent, and effects over three bonds apart are usually negligible [130].

#### 4.1.2. ^13^C-NMR Data Obtained for Caffeoyl Ester Derivatives

When C_3_-OH (or C_4_-OH) undergoes esterification, -OCOCH_3_ is the electron-donating group, which increases the electron cloud density of C-3 and C-2 (or C-4 and C-5) and decreases the value of the chemical shift of this carbon. The α-site of the substituent group is the most influential, followed by the β-site, and the γ-site is shifted to higher fields, which is caused by the γ-effect. In general, the induced effect is negligible for the carbon above the γ-site.

When C_9_-OH undergoes esterification, -O(CH_2_)_n_CH_3_ is an electron-donating group, which increases the electron cloud density of C-9 and C-8 and decreases the chemical shift value of this carbon.

### 4.2. Caffeyltartaric Acid

Table S2 shows the ^13^C-NMR and ^1^H-NMR data of caffeyltartaric acid.

The example is as follows. See Table 9 below.

#### 4.2.1. ^1^H-NMR Data Obtained for Caffeyltartaric Acid

Tartaric acid. ^1^H-NMR (600 MHz, CDCl_3_) δ_H_: 4.34 (1H, H-2,3) [131].

A symmetric structure exists for tartaric acid, with esterification of caffeic acid C9 with OH on tartaric acid C-2′ or C-3′, and elevated C_2′_-H, C_3′_-H chemical shift values.

#### 4.2.2. ^13^C-NMR Data Obtained for Caffeyltartaric Acid

Tartaric acid. ^13^C-NMR (150 MHz, CDCl_3_) δ_C_: 178.0 (C-1, C-4), 75.0 (C-2, C-3) [131].

This carbon chemical shift value decreases when esterification of tartaric acid C_2′_-OH (or C_3′_-OH) and caffeic acid C_9_-OH occurs.

### 4.3. Caffeic Acid Amide Derivatives

Table S3 shows the ^13^C-NMR and ^1^H-NMR data of caffeic acid amide derivatives.

The example is as follows. See Table 10 below.

#### 4.3.1. ^1^H-NMR Data Obtained for Caffeic Acid Amide Derivatives

When the esterification of C_9_-OH occurs, it has little effect on the chemical shift value of H-8 because the induced effect is transmitted through bonding electrons, and the influence of the induced effect diminishes as the distance from the electronegative substituent increases, and the effect of more than three bonds apart is usually negligible.

#### 4.3.2. ^13^C-NMR Data Obtained for Caffeic Acid Amide Derivatives

When C_9_-OH undergoes esterification, which increases the C-9 electron cloud density, the chemical shift value of this carbon decreases.

### 4.4. Caffeoyl Shikimic Acid

Table S4 shows the ^13^C-NMR and ^1^H-NMR data of caffeoyl shikimic acid.

The example is as follows. See Table 11 below.

#### 4.4.1. ^1^H-NMR Data Obtained for Caffeoyl Shikimic Acid

Shikimic acid C_3_-OH, C_4_-OH, and C_5_-OH can be acylated with caffeic acid C_9′_-COOH to form esters. Once the hydroxyl group is ester, the hydrogen signal connected to the same carbon will shift to the low field 1.1~1.6.

#### 4.4.2. ^13^C-NMR Data Obtained for Caffeoyl Shikimic Acid

Shikimic acid C3-OH, C4-OH and C5-OH can be acylated to esters with caffeic acid C9′-COOH, with the carbon (C3, C4, or C5) chemical shift values of the shikimic acid directly linked to the caffeoyl shifted to the low field and the chemical shift values of the caffeoyl C9′ shifted to the high field. Shikimic acid C_3_-OH, C_4_-OH and C_5_-OH can be acylated to esters with caffeic acid C_9′_-COOH, with the carbon (C_3_, C_4_, or C_5_) chemical shift values of the shikimic acid directly linked to the caffeoyl shifted to the low field and the chemical shift values of the caffeoyl C_9′_ shifted to the high field.

### 4.5. Caffeoyl Quinic Acid

Tables S5–S8 show the ^13^C-NMR and ^1^H-NMR data of caffeoyl quinic acid.

The example is as follows. See Table 12 below.

Due to its proximity to H-3 and H-5, H-4 usually appears as a double–double peak. For C4-OH without esterification, H-4 usually occurs between *δ*_H_ 3.7 and 4.1. When C4-OH is esterified, H-4 is displaced to the lower field *δ*_H_ 1.0~1.6. Due to the presence of H-4″ at *δ*_H_ 5.11, compound **41** can be identified as 4-O-caffeoyl-substituted dicaffeoylquinic acids. H-3 (or H-5) generally appears as a multiple peak due to its coupling to H-2 (or H-6) and H-4. For C3-OH (or C5-OH) without esterification, H-3 (or H-5) usually occurs between *δ*_H_ 4.0 and 4.6. When C3-OH (or C5-OH) is esterified, H-3 (or H-5) shifts to a low field of *δ*_H_ 1.0~1.6. The caffeoyl group at the C-3 position is on the upright bond, and H-4 and H-5 maintain the coupling state of the neighboring ax-ax, resulting in a double–double peak at H-4. By observing the signal, compound **41** was identified as 3,4-dicaffeoylquinic acid [43].

#### 4.5.1. ^1^H-NMR Data Obtained for Caffeoyl Quinic Acid

If C_1_-OH is not esterified in the quinic acid parent nucleus, the chemical shifts of H-4 and H-6 are typically between *δ*_H_ 2.1 and 2.3 in the form of multiple peaks. When C_1_-OH is esterified, the resonance frequencies of the four hydrogens in H-2 and H-6 become significantly different, appearing in the hydrogen spectrum as four double–double peaked protons with different chemical shifts (*δ*_H_ 2.0~3.5). Due to its proximity to H-3 and H-5, H-4 usually appears as a double–double peak. For C_4_-OH without esterification, H-4 usually occurs between *δ*_H_ 3.7 and 4.1. When C_4_-OH is esterified, H-4 is displaced to the lower field *δ*_H_ 1.0~1.6. H-3 (or H-5) generally appears as a multiple peak due to its coupling to H-2 (or H-6) and H-4. For C_3_-OH (or C_5_-OH) without esterification, H-3 (or H-5) usually occurs between *δ*_H_ 4.0 and 4.6. When C_3_-OH (or C_5_-OH) is esterified, H-3 (or H-5) shifts to a low field of *δ*_H_ 1.0~1.6 [44].

Once the hydroxyl group becomes an ester, the hydrogen signals attached to the same carbon are shifted to the lower field 1.1~1.6, and the five-position is more significant than the three-position. For molecules with two acylation groups, the shift of the hydrogen signal to the lower field will be more obvious, which may result from mutual accumulation. Regular acylation of caffeoyl quinic acid generally also shifts the two trans-alkene hydrogens (H-7′ and H-8′) on the caffeic acid unit to the low field.

Coupling constants are also important in structural inference, especially in stereo-structural and conformational problems. For example, when the acylating group is in the ax bond, the coupling constants of the two neighboring hydrogens in the eq-eq conformation or the eq-ax conformation are 2~3 Hz. When the acylation group is in the eq bond, the coupling constant of the neighboring ax-ax configuration hydrogen is 10 Hz, and the coupling constant of the eq-ax configuration is 5 Hz [129].

#### 4.5.2. ^13^C-NMR Data Obtained for Caffeoyl Quinic Acid

^13^C NMR showed two carbonyl carbons (*δ*_C_ 165~175). The chemical shifts of C-2 and C-6 in the quinic acid unit are usually between *δ*_C_ 30 and 40. The chemical shifts of C-1, C-3, C-4 and C-5 are within *δ*_C_ 60~80 due to hydroxyl substitution [129].

Quinic acid fragments C_1_-OH, C_3_-OH, C_4_-OH and C_5_-OH can be acylated with caffeic acid to form esters with the presence of acylation shifts, which corresponds to an increase in the chemical shift value of the carbon and a decrease in the caffeoyl C-9′ (or C-9″) chemical shift value. If the chemical shift values of H-2 and H-6 and C-2 and C-6 are very similar, the molecule may have symmetry. When methoxy binds to the C-7 carbonyl group of quinic acid to form an ester, the C-7 carboxyl group shifts to a higher field.

### 4.6. Caffeoyl Danshensu

Tables S9–S11 show the ^13^C-NMR and ^1^H-NMR data of caffeoyl danshensu. As a basis for spectral analysis, the spectral characteristics of two different structural types of compounds, rosmarinic acid and prolithospermic acid, are described below.

The structure of rosmarinic acid (**74**) is characterized by the absence of a substituent at the two-position of the caffeoyl; thus, the aromatic protons of caffeoyl (H-2, 5, 6) are shown as a one, two, four coupling system. A group of danshensu side chain protons in the high field region *δ*_H_ 2.8~5.0 show the spin coupling (ABX, H2-7, H-8) system, which constitutes an important feature of the hydrogen spectrum of these compounds. It is not difficult to find the relevant characteristic peaks from the carbon spectrum. Since the polymerization unit of these compounds has a unit structure containing an o-diphenol hydroxyl group and four oxygenated aromatic quaternary carbons appear in the *δ*_C_ 140~150 interval, the degree of polymerization is two. The CH_2_ peak of *δ*_C_ 38.1 and the CH peak of 78.4 indicate that the dimer contains the structural unit of 3,4-dihydroxyphenyllactic acid.

The structural difference between prolithospermic acid (**91**) and rosmarinic acid is that prolithospermic acid contains a unit structure of dihydrofuran rings. The absolute configurations of the two chiral carbons of the dihydrofuran ring are the R and S configurations. In its high-resolution spectrum, the chemical shifts of a set of characteristic alicyclic hydrogens of the dihydrofuran ring are *δ*_C_ 5.85 and *δ*_C_ 4.31 (H-7, H-8), with a coupling constant of approximately 4.0 Hz in ^1^H-NMR. In ^13^C-NMR, the CH peak is *δ*_C_ 88.0 and the CH peak (C-8) is 57.2.

The spectral characteristics of trimers and tetramers in salvianolic acid are the above two condensation modes. In the analysis of the structure, the degree of polymerization was first determined by the number of oxygenated aromatic season carbons (*δ*_C_ 140~150) in the carbon spectrum, and then the characteristic peak of the high field region in the hydrogen spectrum or carbon spectrum was used to determine the polymerization mode.

Examples are as follows. See Table 13 below.

#### 4.6.1. ^1^H-NMR Data Obtained for Caffeoyl Danshensu

The two benzyl hydrogen signals (*δ*_H_ 3.0~3.5) and the proton signature of the same carbon as the acyloxy group (*δ*_C_ 5.18~5.33) of the danshensu part, and the latter split with the benzyl hydrogen to form a double–double peak *J* = 7 Hz and 4 Hz. The chemical shifts of some aromatic hydrogens in danshensu generally occur at *δ*_H_ 6.7~6.9, and the cleavage is insignificant when the hydroxyl group is methylated.

Usually, when the caffeic acid ester C_9′_-COOH is formed from danshensu C_8_-OH, the chemical shift value of H-8 moves to the low field, and caffeoyl C-2′ and C-3′ are connected with dihydrofuran rings.

#### 4.6.2. ^13^C-NMR Data Obtained for Caffeoyl Danshensu

When the ester of caffeic acid C_9′_-COOH is formed from danshensu C_8_-OH, the C-5, C-6, C-7, C-8 and C-9 chemical shift values shift to the high field, and caffeoyl C-8′ and C-9′ chemical shift values shift to the high field. Caffeoyl C-2′ and C-3′ are linked to the dihydrofuran ring, and the chemical shift values of C-2′ and C-3′ are shifted to the lower domains.

### 4.7. Caffeoyl Glycoside

Tables S12–S18 show the ^13^C-NMR and ^1^H-NMR data of caffeoyl caffeoyl glycoside.

The sugars connected by caffeoyl glycosides are generally approximately 1 to 4. The characteristic end-substrate proton signals at *δ*_H_ 4.3~6.0 and end-substrate carbon signals at *δ*_C_ 95~105 can be used to initially determine the number of sugars. Furthermore, 2D NMR techniques, such as Heteronuclear Multiple Quantum Coherence (HMQC), ^1^H detected heteronuclear multiple bond correlation (HMBC) and total correlation spectroscopy(TOCSY), were used to determine the type of sugar and ascribe the signal for sugar.

The example is as follows. See Table 14 below.

In the HMBC spectra, the correlation between *δ*_H_ 5.03 (H-5″) and *δ*_C_ 169.1 (C-9), *δ*_C_ 60.4 (C-6″), *δ*_C_ 36.9 (C-4″) and *δ*_C_ 103.1 (C-3″) indicates that the caffeoyl group is adjacent to the 5″-OH. In addition, HMBC spectra also showed that the ^1^H NMR signal of *δ*_H_ 5.16 (H-1″) correlated with the ^13^C NMR signal of *δ*_C_ 142.5 (C-2″), *δ*_C_ 67.0 (C-7″), and *δ*_C_ 43.3 (C-8″). The ^1^H NMR signal of *δ*_H_ 4.80 (H-1′) was related to the ^13^C NMR signal of *δ*_C_ 95.2 (C-1″), indicating that the sugar residue is attached to 1″-OH. All ^1^H and ^13^C NMR signals of compound **121** were resolved by ^1^H-^1^H COSY, HSQC and HMBC spectra. The ROESY spectra and coupling constants were analyzed to determine the relative configuration of compound **121**. Based on the large coupling constant (15.6 Hz) between H-7 and H-8, it indicated that the caffeoyl portion is E-configuration. The NMR chemical shift values of compound 121 combined with the GC analysis results of the sugar and D-glucose obtained by acid hydrolysis showed that the hexose part was D-glucose. The high coupling constant (7.8 Hz) from ^3^*J*_H-1′,H-2′_ indicates that the glucosyl unit is β-oriented. Based on the above inferences, compound **121** was identified as Verminoside [90].

#### 4.7.1. ^1^H-NMR Data Obtained for Caffeoyl Glycoside

The type of sugar in caffeoyl glycosides can be determined by the chemical shift and coupling constant observed for the characteristic end-substrate hydrogen signal of the sugar. In general, the end-substrate proton signals of sugar in ^1^H NMR are approximately *δ*_H_ 5.0 ppm, *δ*_H_ 4.3~6.0, 1H(d), glucose *δ*_H_ 4.2~4.4 (d, *J* = 8.0 Hz) and rhamnose *δ*_H_ 5.1~5.3 (d, *J* = 1.0 Hz). Most compounds showed characteristic double peaks, while a few showed wide single peaks. The glycyclic proton signal is between *δ*_C_ 3.5~4.5 ppm. The methyl proton signal of methyl five-carbon sugars (such as rhamnose) is approximately *δ*_H_ 1.0 ppm. The signals of the end-substrate and methyl proton are far away from other signals and can be easily recognized, and the number of sugars, the types of sugars and the location of connections can be inferred.

The relative configuration of the glycoside bond was determined by ^1^H-NMR and the coupling constants of C_1_-H and C_2_-H. In most monosaccharides, such as glucose and their glycosides, the two-sided angle between the end-group proton and H-2 is 180° because H-2 on the sugar is located on the upright bond when the oxygen on the end group is β-oriented, and the ^3^*J*_H1,H2_ value is approximately 6~8 Hz. For the α-configuration, the angle between the two surfaces is 60°, and the ^3^*J*_H1,H2_ values are from 1 to 3 Hz. The terminal group configuration of pyranose with H-2 in the upright bond can be determined by the ^3^*J*_H1,H2_ values of the terminal group hydrogen measured by ^1^H-NMR spectra. However, in rhamnoside, differentiation through the ^3^*J*_H1,H2_ values is impossible because H-2 is located on the flat-volume bond, and the dihedral angles of the two protons are 60° in both the α and β configurations of the end group. For furanose, regardless of whether its end matrix and C_2_ proton are in cis or trans, its *J* value does not change much (the value remains in 0~5), so the glycoside bond configuration cannot be judged.

#### 4.7.2. ^13^C-NMR Data Obtained for Caffeoyl Glycoside

##### Type and Amount of Sugar

The diversity of caffeoyl glycosides is evidenced by the type of glycosides and the sugar fraction, as there are differences in the number of sugars, the types of sugars, the way the sugars are connected to each other and the way the sugars are connected to the glycosides.

The chemical shift of the methyl carbon of the sugar is around *δ*_C_ 18, and the presence of multiple signals (minus the methyl group in the glycoside) can indicate the presence of several methyl pentoses. CH_2_OH is approximately *δ*_C_ 62, and CHOH is approximately *δ*_C_ 68~85. The carbon signal in the furanose ring appears in a lower field than that in the pyranose ring, which can distinguish the size of the sugar–oxygen ring. For the furan oxygen ring, CH-OH (C_3_, C_5_) >80 ppm; for the pyran oxygen ring, CH-OH (C_3_, C_5_) <78 ppm. Most of the end-group carbon signals of glycosides are between 95 and 105, such as glucose and rhamnose with *δ*_C_ 105.1 and *δ*_C_ 103.8, respectively. Several signals can indicate the presence of several sugars in the repeating units of the sugar chain; most of the signals on the sugar can be specified by comparison with similar sugars or glycoside derivatives.

The end-group differential isomers of glycosides, such as glucose, leading to large differences in the chemical shift values of the end-group carbons, and the relative configuration (α or β) of the sugar can be determined from the chemical shift values of the end-group carbons. In common sugars, the end-group carbonization shift of β-D and α-L glycosides is usually greater than *δ*_C_ 100. When ester glycosides, tertiary alcohol glycosides, and individual phenolic glycosides are present, the chemical shift values can drop to *δ*_C_ 98. The end group carbon chemical shift values for α-D- and β-L-type glycosidic bonds are usually less than *δ*_C_ 100. Therefore, the number of sugars and the conformation of glycosidic bonds contained in oligosaccharides and glycosides can be roughly inferred from the number of carbon signals and chemical shift values in the *δ*_C_ 95~105 region [132].

##### Determining the Binding Position of Sugar (the Glycosylation Position)

Currently, ^13^C NMR methods are often used to determine the location of sugar linkages in caffeoyl glycosides, which primarily involves attributing signals to individual carbons to identify the carbon that produces the glycosidic shift. In practical work, the attribution of chemical shifts is mainly based on comparison with analogs and reasonable prediction by the rule of glycosylation shift, and the selected reference compounds are generally free glycosides and methyl glycosides.

The linkage between sugars and aglycones in caffeoyl glycosides is formed by the combination of the hydroxyl groups of sugars and aglycones. The carboxyl group of sugar and aglycone combine to form an ester bond. In hydroxyl glycosylation, C generally shifts *δ*_C_ 8 to 10 toward the lower field, and it affects the values of neighboring C. Glycosylation of the link position between sugars generally moves the shift to the low field at approximately *δ*_C_ 3~8. However, sugars form ester glycosides with carboxyl groups, the glycosylation shift value is high, the carboxyl carbon glycosidic shift is approximately two, and the end group carbon of the sugar is generally shifted to *δ*_C_ 95~96. When sugars form glycosides with carboxyl groups, phenolic hydroxyl groups and enol hydroxyl groups, the glycosylation shift value is relatively special, the α-C shift to the high field is 0~4 units, and the β-C shift to the low field direction. The sugar end-group carbon is displaced to the low field in phenolic and enol glycosides and the high field in ester glycosides, with small displacements (0~4 units). Typically, acetylation of the hydroxyl group shifts its alkyl carbon (α-C) signal to the low field (+2~+4 ppm) and its neighboring carbon (β-C, which is γ-C with respect to the acetyl group) signal to the high field (−6~−2 ppm).

To determine the position of the linkage between the two monosaccharides in a disaccharide glycoside, the ^13^C spectral data of the disaccharide glycoside were compared with the ^13^C spectral data of the corresponding monosaccharide. If the chemical shift of a carbon atom of the inner sugar is shifted in the low-field direction (usually 4~7 ppm) and the chemical shifts of its two neighboring carbon atoms are slightly shifted in the high-field direction (approximately 1~2 ppm), this carbon atom of the inner sugar is the linkage position of the sugar.

To identify the signals of individual carbon and H atoms, spectroscopic techniques such as HMBC and nuclear overhauser effect spectroscopy (NOESY) were utilized to infer the linkage order and linkage position of the sugar chain by observing the linked CH or HH remote coupling.

## 5. Possible Biosynthetic Pathways for the Generation of Caffeic Acid and Its Derivatives

Basically, most of the phenolics in higher plants are synthesized by the mangiferic acid pathway. Carbon dioxide in plant photosynthesis forms primary carbon metabolites, glucose and some other carbohydrates. These primary metabolites are generated through glycolysis and other ways to generate erythrose and phosphenol-pyruvate through the catalytic conversion of related enzymes into shikimic acid and then shikimic acid into phenylalanine, tyrosine, tryptophan and other aromatic amino acids [133]. Phenylalanine generates cinnamic acid by the action of phenylalanin ammonia-lyase (PAL), which in turn generates 4-coumaric acid by the action of cinnamic acid 4-hydroxylase (C4H) and the production of 4-coumaroyl-CoA by the action of 4-coumarate:coenzyme a ligase (4CL) [134,135]. C3H catalyzes the formation of caffeic acid from coumaric acid [136].

The specific biosynthetic pathways contain the following main pathways: first, rosmarinic acid and salvianolic acids are synthesized from 4-coumaric acid and 4-hydroxyphenyllactic acid as precursors; second, chlorogenic acids are synthesized from quinic acid and caffeic acid as precursors; and third, caffeic acid, tyrosol and hydroxytyrosol are used as precursors to synthesize caffeoyl glycosides, such as acteoside (Figure 1).

### 5.1. Caffeoyl Ester Derivatives, Caffeyltartaric Acid, Caffeic Acid Amide Derivatives, Caffeoyl Shikimic Acid and Caffeoyl Quinic Acid

There are three biosynthetic pathways by which 4-coumaroyl-CoA continues to produce chlorogenic acid (CGA), and these pathways are still debated. The following biosynthetic pathways have been proposed: the first pathway is that hydroxycinnamoyl CoA shikimate hydroxycinnamoyl transferase (HCT) can catalyze the hydroxylation of 4-coumaroyl-CoA to react with shikimic acid to produce 4-coumaroyl shikimic acid ester, which further generates caffeoyl shikimic acid, and finally, caffeoyl-CoA, and hydroxycinnamoyl CoA quinate hydroxycinnamoyl transferase (HQT) can catalyze caffeoyl-CoA and quinic acid to synthesize CGA through transesterification. The second pathway suggests that CGA is derived from quinic acid and caffeoyl D-glucose and is catalyzed by hydroxycinnamoyl D-glucose: quinate hydroxycinnamoyl transferase (HCGQT). In the third pathway, p-coumaroyl quinic acid is produced through catalyzed by HCT and then CGA is produced by p-coumarate 3-hydroxylase (C3H) hydroxylation [137,138].

The biosynthesis of chicoric acid involves a two-step process. In the cytosol, two BAHD acyltransferases, EpHTT and EpHQT catalyze the production of caftaric acid and chlorogenic acid intermediates, respectively. Both compounds are transported to the vacuole to form chicoric acid catalyzed by EpCAS [139].

However, the biosynthetic pathway that generates caffeoyl ester derivatives and caffeic acid amide derivatives in nature is not well understood.

### 5.2. Caffeoyl Danshensu

The caffeoyl danshensu biosynthesis pathway includes two parallel pathways, the phenylalanine pathway and the tyrosine pathway. The tyrosine of the tyrosine branch is treated by tyrosine aminotransferase (TAT) to produce 4-hydroxyphenylpyruvic acid, and 4-hydroxyphenylpyruvic acid is treated by 4-hydroxyphenylpyruvate reductase (HPPR) to produce 4-hydroxyphenylpyruvic acid. Biochemical studies have shown that the initial stage of rosmarinic acid (RA) in Salvia miltiorrhiza is the hydroxylation of 4-hydroxyphenyllactic acid (pHPL) at aromatic ring C-3, which is catalyzed by an unknown CYP450 to produce 3,4-dihydroxyphenyllactic acid (DHPL) [140]. Rosmarinic acid synthase (RAS) then binds DHPL to the 4-coumaroyl portion to form the ester 4-coumaroyl-3,4-dihydroxyphenyllactic acid, which is hydroxylated by the cytochrome p450-dependent monooxygenase CYP98A14 to form RA. It differs from parts of other plants, where pHPL is a direct substrate of RAS, bound to 4-coumaryl-coa, and RA is formed by the dihydroxylation of esters [141,142,143,144,145]. RA is formed by the hydroxylation of 3-hydroxylase (3-H) and 3′ hydroxylase (3′-H) [141,146,147,148].

Caffeic acid is catalyzed to form caffeoyl-CoA, which is then catalyzed by RAS with 4-hydroxyphenyllactic acid to form caffeoyl-4′-hydroxyphenyllactic acid and then catalyzed by CYP98A14 to form RA [140].

The biosynthetic pathway from rosmarinic acid to salvianolic acid B is still not fully understood. However, in a study by Di et al. [140], the following synthetic route was suggested: salvianolic acid B is produced by direct polymerization of two molecules of rosmarinic acid, which involves a redox reaction catalyzed by an unknown oxidase. After performing a comprehensive analysis of key enzyme-encoding genes in the biosynthesis pathway of active ingredients in salvianolic acid, Xu et al. [149] found that five genes encoding laccases were detected in the biosynthesis pathway of salvianolic acid. Among them, two genes are closely related to the content of salvianolic acid and other macromolecules, such as salvianolic acid B. Therefore, they speculate that the process of rosmarinic acid synthesis of salvianolic acid is likely to be catalyzed by laccase in *Salvia miltiorrhiza* [11].

### 5.3. Caffeic Acid Glycoside

Acteoside is among the most widely distributed disaccharide caffeoyl esters, consisting of the following components: CA, glucose, rhamnose and hydroxytyrosol (3,4-dihydroxyphenylethanol, HT). At present, there is a general consensus on the potential metabolic modules of acteoside biosynthesis, which mainly include the phenylalanine metabolic pathway, dopamine pathway/tyramine pathway and downstream acyl transfer and glycosylation crossing pathway.

The intermediates of the dopamine/tyramine pathway, tyrosol and hydroxytyrosol, are another key precursor to the biosynthesis of acteoside. Both intermediates can generate hydroxytyrosol glucoside, which is the precursor of acteoside and can be produced through different pathways, which is an important branch pathway of acteoside biosynthesis. Tyrosine produces L-DOPA by polyphenol oxidase (PPO)/tyrosine hydroxylase (TH), and DOPA decarboxylase (DODC)/tyrosine decarboxylase (TyDC) catalyzes the production of dopamine from L-DOPA, which is later followed by hydroxytyrosol by the action of copper amine oxidase (CuAO) and alcohol dehydrogenase (ALDH) [150,151,152,153,154]. In the other pathway, dopamine is catalyzed by copper amine oxidase (CuAO)/monoamine oxidase (MAO) to generate 3,4-dihydroxyphenylpyruvic (3,4-DHPAA), after which ALDH catalyzes the generation of hydroxytyrosol from 3,4-DHPAA [153]. The tyramine pathway can provide tyrosol or hydroxytyrosol precursors for the acteoside biosynthesis pathway. Tyrosine is catalyzed by TyDC to form tyramine, which is oxidized by CuAO/tyramine oxidase (TYO) to 4-hydroxyphenylacetaldehyde (4-HPAA). 4-HPAA can be reduced to tyrosol by 4-hydroxyphenylpyruvate reductase (4HPAR)/ALDH, which then generates hydroxytyrosol catalyzed by tyrosol hydroxylase (TLH) [150,151,153,155,156].

4-Hydroxyphenylpyruvic acid (4-HPPDC) generates 4-HPAA by the action of 4-hydroxyphenylpyruvate decarboxylase (HPPADC) [157,158]. Torrens Spence et al. [159] first identified pyridoxal phosphate-dependent 4-hydroxyphenylacetaldehyde synthase (4HPAAS), which directly catalyzes the conversion of tyrosine to 4-HPAA.

Based on the structural analogs of the acteoside, their centers are glucose, esterified with caffeoyl groups and modified by rhamnosyl groups at the C3 position, but the molecular catalytic mechanism leading to their acylation and rhamnosylation has not been reported. From the hydrolysis, metabolism experiments and chemical structure of the acteoside, it can be inferred that there are two potential possibilities for the synthesis of acteoside [160,161,162]. First, caffeoyl-CoA and hydroxytyrosol glucoside generate derhamnosylacteoside under the action of HCT, and derhamnosylacteoside is catalyzed by UDP-rhamnose glucosyltransferase (URT) to further generate acteoside. Another potential pathway is that hydroxytyrosol generates hydroxytyrosol glucoside by UDP-glucose:glycosyltransferase (UGT), hydroxytyrosol glucoside is catalyzed by URT to generate dercaffeoylacteoside, and dercaffeoylacteoside is finally condensed with caffeoyl-CoA to generate acteoside by the action of HCT.

## 6. Conclusions

Caffeic acid and its derivatives are widely distributed in plants, exhibit many physiological activities, undergo rapid metabolism, have a relatively simple chemical structure and are natural active ingredients with good application prospects. In this paper, the structural information and NMR data of 1743 caffeic acid and its derivatives are reviewed and compiled to summarize the patterns of chemical shifts and the effects of their neighboring and interstitial substituents for the seven major classes of compounds. From the statistical results, in general, the acetylation of the hydroxyl group will shift its alkyl carbon (α-C) signal to the low field (+2~4 ppm) and its neighboring carbon (β-C, relative to the γ-C of the acetyl group) signal to the high field (−6~−2 ppm).

After the sugar is glycosidized with a glycoside, the chemical shift values of the α-C and β-C of the glycoside and the end-group carbons of the sugar are changed, and this change is called a glycosidization shift. The value of the glycosylation shift is related to the structure of the aglycone but not to the type of sugar. If the aglycone is a chain structure, the glycosidation shift value of the sugar end group carbon decreases as the glycosidic element is a primary, secondary or tertiary group. In the structure of the glycosidized sugar molecule, the chemical shift of α-C, which is usually directly connected to the end-group carbon, is more varied, and β-C is slightly affected, while other carbon atoms are less affected. When sugar and alcohol hydroxyl groups form glycosides, the sugar end group carbon shifts to a lower field and the displacement amplitude is related to the type of alcohol of the aglycone.

The ^13^C-NMR and ^1^H-NMR shift characteristics observed for different substitution types of caffeic acid and its derivatives can provide a basis and reference for the identification of caffeic acid and its derivatives in the future; in addition, this information provides predictions for the discovery of new structures and strong evidence for the study of metabolites of caffeic acid and its derivatives in vivo. A 2D NMR spectroscopy plays an important role in determining the structure of new phenolic acids. The application of C,H-COSY and C,H-COLOC enables rapid structural determinations of new compounds and more accurate attribution of chemical shifts to individual protons and carbons.

The biosynthetic pathways of caffeic acid and its derivatives are also summarized in this paper. Both caffeic acid and its derivatives are first synthesized in plants through the shikimic acid pathway, from which phenylalanine is deaminated to cinnamic acid and converted to caffeic acid. The specific biosynthetic pathways contain the following main pathways: first, rosmarinic acid and salvianolic acids are synthesized from 4-coumaric acid and 4-hydroxyphenyllactic acid as precursors; second, chlorogenic acids are synthesized from quinic acid and caffeic acid as precursors; and third, caffeic acid, tyrosol and hydroxytyrosol are used as precursors to synthesize caffeoyl glycosides such as acteoside.

However, as methods to effectively mine gene elements and gene function identification methods are lacking, progress in the analysis of caffeic acid and its derivatives has been slow, and its biosynthetic pathway has not been fully elucidated. Based on the above progress achieved for the biosynthetic pathways of caffeic acid and its derivatives, the biosynthetic pathway of chlorogenic acid-like components is relatively clear. The biogenic pathway of salvianolic acids is not completely clear, and only the upstream rosmarinic acid biogenic pathway has been partially clarified. The source pathway of other phenolic acids downstream of rosmarinic acid, such as salvianolic acid B, is not completely clear and is only in the preliminary stage of exploration and speculation. Although laccase has been identified and hypothesized to play an important role in the biosynthetic pathway of salvianolic acid B, the gene for laccase has not been completely cloned or validated. Therefore, it is necessary to continue exploring the role of laccase in catalyzing rosmarinic acid synthesis of salvianolic acid B and elucidate its mechanism. The key elements and pathways of acteoside synthesis have not been fully resolved, mainly acyltransferase and rhamnosyltransferase in the downstream acyltransferase and glycosylation pathways have not been reported, and the catalytic sequence of acylation and rhamnosylation has not been verified, which has hindered the biosynthesis of acteoside and requires further exploration.

At present, much research on caffeic acid derivatives is being carried out, and researchers are trying to find new caffeic acid derivative drugs with richer biological activities. With the deepening of research, the biosynthetic pathway system of caffeic acid and its derivatives will be increasingly clarified, and more natural drugs or synthetic drugs will be developed based on the caffeic acid derivative family.

## Figures and Tables

**Table 1:** Caffeic acid and caffeoyl ester derivatives.

No.	Compound Name	CAS Registry Number	Reference
**1**	Caffeic acid	331-39-5	[12]
**2**	methyl caffeate	3843-74-1	[13]
**3**	Ethyl-Caffeate	102-37-4	[14]
**4**	3-O-Acetylcaffeic acid	134840-99-6	[15]
**5**	4-O-Acetylcaffeic acid	260976-59-8	[16]
**6**	trans-Caffeic acid octyl ester	478392-41-5	[17]
**7**	trans-Caffeic acid stearyl ester	69573-60-0	[18]
**8**	Eicosanyl (E)-caffeate	28593-90-0	[19]
**9**	trans-Caffeic acid docosanyl ester	28593-92-2	[20]
**10**	Caffeic acid 3,4-dihydroxyphenethyl ester	918434-42-1	[21]

**Table 2:** Caffeyltartaric acids.

No.	Compound Name	CAS Registry Number	Reference
**11**	Caftaric acid	67879-58-7	[22]
**12**	Chicoric acid	6537-80-0	[23]
**13**	2-O-caffeoyl-3-O-isoferuloyltartaric	2704534-41-6	[24]
**14**	2-O-caffeoyl-3-O-feruloyltartaric acid	99119-75-2	[24]
**15**	2-O-caffeoyl-3-O-p-coumaroyltartaric acid	116064-67-6	[22]
**16**	2-O-caffeoyl-3-O-5[-α-carboxy-β-(3,4-dihydroxyphenyl) ethyl] caffeoyltartaric	117841-80-2	[25]
**17**	2,3-O-di-5[α-carboxy-β-(3,4-dihydroxyphenyl) ethyl] caffeoyltartaric acid	117869-66-6	[25]

**Table 3:** Caffeic acid amide derivatives.

No.	Compound Name	CAS Registry Number	Reference
**18**	N-caffeoyl-phenylalanine	170663-16-8	[26]
**19**	phaselic acid	860295-20-1	[27]
**20**	N-[(2E)-3-(3,4-Dihydroxyphenyl)-1-oxo-2-propen-1-yl]-L-glutamic acid	860295-23-4	[27]
**21**	trans-Clovamide	53755-02-5	[27]
**22**	Caffeoyl-N-tyrosine	124027-56-1	[27]
**23**	Pelliolatifolia A	798568-99-7	[28]
**24**	Pelliolatifolia B	/	[28]
**25**	Pelliolatifolia C	/	[28]

**Table 4:** Caffeoyl shikimic acids.

No.	Compound Name	CAS Registry Number	Reference
**26**	5-O-Caffeoylshikimic acid	73263-62-4	[29]
**27**	4-O-Caffeoylshikimic acid	180842-65-3	[30]
**28**	3-O-Caffeoylshikimic acid	180981-12-8	[31]

**Table 5:** Caffeoyl quinic acids.

No.	Compound Name	CAS Registry Number	Reference
**29**	Chlorogenic acid	327-97-9	[32]
**30**	Methyl chlorogenate	29708-87-0	[33]
**31**	Ethyl chlorogenate	37123-66-3	[34]
**32**	Butyl chlorogenate	132741-56-1	[35]
**33**	Cryptochlorogenic acid	905-99-7	[32]
**34**	4-O-Caffeoylquinic acid methyl ester	123372-74-7	[36]
**35**	Neochlorogenic acid	906-33-2	[37]
**36**	5-O-Caffeoylquinic acid methyl ester	123410-65-1	[36]
**37**	1-Caffeoylquinic acid	1241-87-8	[38]
**38**	Isochlorogenic acid a	2450-53-5	[37]
**39**	3,5-Di-O-caffeoylquinic acid methyl ester	141545-93-9	[39]
**40**	3,5-O-Dicaffeoylquinic acid ethyl ester	143051-74-5	[34]
**41**	Isochlorogenic acid b	14534-61-3	[37]
**42**	3,4-O-Dicaffeoyl quinic acid methyl ester	114637-83-1	[39]
**43**	Isochlorogenic acid c	57378-72-0	[37]
**44**	4,5-O-Dicaffeoyl quinic acid methyl ester	188742-80-5	[40]
**45**	1,3-Dicaffeoylquinic acid	19870-46-3	[41]
**46**	1,4-Dicaffeoylquinic acid	1182-34-9	[42]
**47**	1,5-Dicaffeoylquinic acid	30964-13-7	[43]
**48**	1,3,5-Tricaffeoylquinic acid	1073897-80-9	[44]
**49**	3,4,5-Tricaffeoylquinic acid	437611-66-0	[45]
**50**	1,5-Dicaffeoyl-3-succinoylquinicacid	438238-31-4	[46]
**51**	1,5-Dicaffeoyl-4-succinoylquinicacid	1073898-11-9	[46]
**52**	1,5-Dicaffeoyl-3,4-disuccinoylquinic acid	1073898-12-0	[47]
**53**	4-syringoy-5-Caffeoylquinic acid	1207645-15-5	[48]
**54**	3-syringoy-4-Caffeoylquinic acid	1207645-17-7	[48]
**55**	3-syringoy-5-Caffeoylquinic acid	1207645-16-6	[48]
**56**	3-syringoy-5-Caffeoylquinic acid methyl ester	946599-45-7	[49]
**57**	3-sinapoyl-4-Caffeoylquinic acid methyl ester	1820924-08-0	[49]
**58**	3-sinapoyl-5-Caffeoylquinic acid methyl ester	879627-83-5	[50]
**59**	4-sinapoyl-3-Caffeoylquinic acid methyl ester	879627-81-3	[51]
**60**	3-vanilloyl-4-Caffeoylquinic acid	1819972-60-5	[51]
**61**	4-vanilloyl-5-Caffeoylquinic acid	1819972-65-0	[50]
**62**	3-vanilloyl-4-Caffeoylquinic acid methyl ester	2410794-76-0	[50]
**63**	3-vanilloyl-5-Caffeoylquinic acid	1819972-62-7	[50]
**64**	3-vanilloyl-5-Caffeoylquinic acid methyl ester	1819972-63-8	[50]
**65**	4-(7S,8R)-glycosmisoyl-5-Caffeoylquinic acid	1819972-67-2	[50]
**66**	4-(7S,8R)-glycosmisoyl-5-Caffeoylquinic acid methyl ester	1819972-66-1	[50]
**67**	3-(7S,8R)-glycosmisoyl-4-Caffeoylquinic acid	1819972-68-3	[50]
**68**	3-(7S,8R)-glycosmisoyl-4-Caffeoylquinic acid methyl ester	1819972-69-4	[50]
**69**	3-dihydrophaseicoyl-5-Caffeoylquinic acid	2230901-33-2	[50]
**70**	4-dihydrophaseicoyl-5-Caffeoylquinic acid	2230901-34-3	[52]
**71**	4-O-(E)-Caffeoyl-5-O-malonylquinic acid	1325713-71-0	[52]

**Table 6:** Caffeoyl danshensu.

No.	Compound Name	CAS Registry Number	Reference
**72**	Danshensu	76822-21-4	[53]
**73**	Salvianolic acid F	158732-59-3	[54]
**74**	Rosmarinic acid	20283-92-5	[55]
**75**	Methyl rosmarinate	99353-00-1	[55]
**76**	Ethyl rosmarinate	174591-47-0	[55]
**77**	Rosmarinic acid-4-O-β-D-glucoside	251926-06-4	[56]
**78**	Rosmarinic acid-3-O-glucoside	178895-25-5	[57]
**79**	Salvianolic acid D	142998-47-8	[58]
**80**	Salvianolic acid A	96574-01-5	[59]
**81**	Methyl salvionolate A	1015171-69-3	[60]
**82**	Isosalvianolic acid A	634583-97-4	[61]
**83**	Salvianolic acid C	115841-09-3	[62]
**84**	Methyl salvianolate C	866011-55-4	[63]
**85**	Salvianolic acid N	933776-37-5	[64]
**86**	Isosalvianolic acid C	142115-17-1	[65]
**87**	Salvianolic acid L	389065-74-1	[66]
**88**	Lithospermic acid A	28831-65-4	[67]
**89**	Monomethyl lithospermate	933054-33-2	[63]
**90**	Dimethyl lithospermate	54844-34-7	[63]
**91**	Prolithospermic acid	145554-86-5	[68]
**92**	Salvianolic acid B	121521-90-2	[59]
**93**	Salvianolic acid Y	1638738-76-7	[69]
**94**	9′′′-Methyl salvianolate B	1167424-32-9	[70]
**95**	Ethyl Salvianolate B	1061341-92-1	[71]
**96**	Salvianolic acid E	142998-46-7	[72]
**97**	Salvianolic acid T	1644284-92-3	[73]
**98**	Salvianolic acid U	2377568-67-5	[73]
**99**	Salvianolic acid J	159736-38-6	[74]
**100**	Salvianolic acid K	203733-40-8	[75]
**101**	Salvianolic acid H	444179-57-1	[76]
**102**	Salvianolic acid I	150072-80-3	[72]

**Table 7:** Caffeoyl glycosides.

No.	Compound Name	CAS Registry Number	Reference
**103**	Caffeic acid 3-(β-1-glucoside)	24959-81-7	[77]
**104**	(E)-Caffeic acid 4-O-β-D-glucopyranoside	147511-61-3	[78]
**105**	6-O-(E)-Caffeoyl-β-D-glucopyranose	209797-79-5	[79]
**106**	1-O-(E)-Caffeoyl-β-D-glucopyranose	13080-40-5	[78]
**107**	Calceolarioside B	105471-98-5	[80]
**108**	Calceolarioside A	84744-28-5	[80]
**109**	Plantainoside A	136172-59-3	[81]
**110**	Plantainoside B	136083-85-7	[81]
**111**	Robustaside B	136172-60-6	[82]
**112**	Plantasioside	163633-33-8	[83]
**113**	Calceolarioside D	114217-05-9	[84]
**114**	6′′-O-caffeoylharpagide	1147125-45-8	[85]
**115**	Callicoside A	2070860-90-9	[86]
**116**	Isonuomioside	135463-05-7	[87]
**117**	Paucifloside	151513-65-4	[88]
**118**	Pubescenoside A	850878-24-9	[89]
**119**	Pubescenoside B	850878-25-0	[89]
**120**	Callicoside B	2070860-91-0	[86]
**121**	Verminoside	50932-19-9	[90]
**122**	Nudifloside	1242769-92-1	[91]
**123**	1,6-Di-O-caffeoyl-β-D-glucopyranoside	23284-22-2	[79]
**124**	Agnucastoside C	610786-31-7	[92]
**125**	Plantainoside D	147331-98-4	[81]
**126**	Plantamoside	104777-68-6	[93]
**127**	Hellicoside	132278-04-7	[94]
**128**	Scrocaffeside A	1034143-61-7	[95]
**129**	Scrocaffeside B	1034143-62-8	[95]
**130**	Scrocaffeside C	1034143-63-9	[95]
**131**	Swertiamacroside	128585-97-7	[96]
**132**	Cistanoside F	97411-47-7	[97]
**133**	Peiioside A1/A2	1610618-94-4	[98]
**134**	Jionoside C	120406-33-9	[99]
**135**	Syringalide A 3′-α-l-rhamnopyranoside	110327-00-9	[100]
**136**	Forsythoside A	79916-77-1	[101]
**137**	Forsythoside H	1178974-85-0	[101]
**138**	Isoforsythiaside	1357910-26-9	[102]
**139**	Forsythoside I	1177581-50-8	[101]
**140**	Isoverbascoside	61303-13-7	[103]
**141**	4′′′-Acetyl-O-isoverbascoside	1885100-32-2	[104]
**142**	3′′′,4′′′-Diacetyl-O-isoverbascoside	1885100-33-3	[104]
**143**	Verbascoside	61276-17-3	[103]
**144**	Cistanoside C	94492-22-5	[105]
**145**	Jionoside D	120406-34-0	[99]
**146**	2′-Acetylacteoside	94492-24-7	[105]
**147**	β-Hydroxyverbascoside	109279-13-2	[106]
**148**	Campneoside II	95587-86-3	[97]
**149**	Forsythoside C	84213-44-5	[107]
**150**	Suspensaside B	251443-71-7	[107]
**151**	β-Oxoacteoside	149507-92-6	[108]
**152**	Lianqiaoxinoside B	1351289-13-8	[109]
**153**	Orobanchoside	61276-16-2	[110]
**154**	Suspensaside A	251443-70-6	[107]
**155**	Samioside	360768-68-9	[111]
**156**	Betonyoside F	181301-33-7	[112]
**157**	Pedicularioside A	135010-61-6	[113]
**158**	Lysionotoside	210837-35-7	[114]
**159**	Forsythoside B	81525-13-5	[115]
**160**	Acetyl forsythoside B	1000071-23-7	[115]
**161**	Lunariifolioside	802972-97-0	[116]
**162**	Marruboside	444105-70-8	[117]
**163**	Calceolarioside C	114217-04-8	[118]
**164**	Forsythoside J	1178975-00-2	[101]
**165**	Raduloside	1165742-60-8	[119]
**166**	Echinacoside	82854-37-3	[120]
**167**	Cistanoside A	93236-42-1	[121]
**168**	Verpectoside B	306287-87-6	[122]
**169**	Poliumoside	94079-81-9	[123]
**170**	Forsythoside F	94130-58-2	[124]
**171**	Isolavandulifolioside	159354-69-5	[125]
**172**	Lavandulifolioside	117895-00-8	[126]
**173**	Chionoside F	1254045-65-2	[127]

**Table 8:** ^1^H and ^13^C NMR Data of compounds **1** and **2** (*δ* in ppm, *J* in Hz).

Position	Caffeic Acid (1) ^a^		Methyl-Caffeate (2) ^b^	
	^1^H (400 MHz)	^13^C (100 MHz)	^1^H (500 MHz)	^13^C (125 MHz)
1	-	127.7	-	127.7
2	7.04 (d, 2.0)	115.1	7.03 (d, 2.0)	115.1
3	-	146.8	-	146.8
4	-	149.4	-	149.5
5	6.75 (d, 8.0)	116.5	6.76 (d, 8.2)	116.5
6	6.93 (dd, 2.0, 8.0)	122.8	6.94 (dd, 8.2,2.0)	122.9
7	7.57 (d, 16.0)	147.1	7.52 (d, 16.0)	146.9
8	6.31 (d, 16.0)	115.5	6.24 (d, 16.0)	114.8
9	-	171.1	-	169.8
10			3.75 (s)	52.0

^a^ Data of **1** were from reference [12] and recorded in CD_3_OD. ^b^ Data were from reference [13] and recorded in CD_3_OD.

**Table 9:** ^1^H and ^13^C NMR Data of compound **11** (*δ* in ppm, *J* in Hz).

Position	Caftaric Acid (11)		Position	Caftaric Acid (11)	
	^1^H (300 MHz)	^13^C (75.5 MHz)		^1^H (300 MHz)	^13^C (75.5 MHz)
1	-	128.04	1′	-	172.46
2	7.10 (d, 1.8)	117.43	2′	5.60 (d, 2.2)	71.54
3	-	145.49	3′	4.91 (d, 2.2)	75.25
4	-	148.64	4′	-	175.08
5	6.87 (d, 8.8)	114.26			
6	7.03, 7.05 (dd, 8.8, 1.8)	116.49			
7	7.62 (d, 15)	148.90			
8	6.33 (d, 15)	124.33			
9	-	169.40			

Data were from reference [22] and recorded in D_2_O.

**Table 10:** ^1^H and ^13^C NMR Data of compound **18** (*δ* in ppm, *J* in Hz).

Position	N-Caffeoyl-Phenylalanine (18)		Position	N-Caffeoyl-Phenylalanine (18)	
	^1^H (400 MHz)	^13^C (100.5 MHz)		^1^H (400 MHz)	^13^C (100.5 MHz)
1	-	129.7	1′	-	135.1
2	6.69 (d, 2.0)	121.2	2′	7.53 (br d, 8.0)	128.7
3	-	145.0	3′	7.33–7.38 (m)	129.7
4	-	146.0	4′	7.33–7.38 (m)	130.7
5	6.67 (d, 8.1)	117.4	5′	7.33–7.38 (m)	129.7
6	6.56 (dd, 8.0, 1.9)	121.7	6′	7.53 (br d, 8.0)	128.7
7	6.65 (d, 15.8)	142.3	7′	2.91 (dd, 8.3, 14.0), 3.10 (dd, 5.2, 14.0)	37.8
8	7.49 (d, 15.9)	116.4	8′	4.72 (dd, 5.3, 8.3)	55.5
9	-	168.3	9′	-	175.0

Data were from reference [26] and recorded in MeOH-*d*_4_.

**Table 11:** ^1^H and ^13^C NMR Data of compound **26** (*δ* in ppm, *J* in Hz).

Position	5-O-Caffeoylshikimic Acid (26)		Position	5-O-Caffeoylshikimic Acid (26)	
	^1^H (500.13 MHz)	^13^C (125.77 MHz)		^1^H (500.13 MHz)	^13^C (125.77 MHz)
1	-	125.9	1′	-	128.5
2	7.00	114.7	2′	6.70	138.6
3	-	145.2	3′	4.30	65.5
4	-	149.7	4′	3.78	68.3
5	6.26	116.4	5′	4.48	70.5
6	6.78	121.6	6′	2.67, 2.19	27.5
7	7.06	149.5	7′	-	168.0
8	5.12	114.9			
9	-	166.7			

Data were from reference [29] and recorded in DMSO-*d*_6_.

**Table 12:** ^1^H and ^13^C NMR Data of compound **41** (*δ* in ppm, *J* in Hz).

Position	Isochlorogenic Acid b (41)		Position	Isochlorogenic Acid b (41)	
	^1^H (500 MHz)	^13^C (125 MHz)		^1^H (500 MHz)	^13^C (125 MHz)
1/1′	-	127.7, 127.7	1″	-	76.1
2/2′	7.03, 7.00 (d, 2.0)	115.3, 115.2	2″	2.26 (m)	39.4
3/3′	-	146.8, 146.8	3″	5.62 (m)	69.0
4/4′	-	149.7, 149.7	4″	5.11 (dd, 9.1, 3.5)	75.8
5/5′	6.75, 6.73 (d, 8.1)	116.5, 116.4	5″	4.37 (m)	69.4
6/6′	6.93, 6.90 (dd, 8.1, 2.0)	123.1, 123.0	6″	2.32, 2.17 (m)	38.4
7/7′	7.60, 7.53 (d, 16.0)	147.7, 147.6	7″	-	176.8
8/8′	6.29, 6.18 (d, 16.0)	114.8, 114.7			
9/9′	-	168.5, 168.2			

Data were from reference [43] and recorded in CD_3_OD.

**Table 13:** ^1^H and ^13^C NMR Data of compounds **74** and **91** (*δ* in ppm, *J* in Hz).

Position	Rosmarinic Acid (74) ^a^		Prolithospermic Acid (91) ^b^		Position	Rosmarinic Acid (74) ^a^		Prolithospermic Acid (91) ^b^	
	^1^H (500 MHz)	^13^C (125 MHz)	^1^H	^13^C		^1^H (500 MHz)	^13^C (125 MHz)	^1^H	^13^C
1	-	128.12	-	124.3	1′	-	131.29	-	133.5
2	7.03 (d, 2.0)	115.27	-	127.4	2′	6.77 (d, 2.0)	117.63	6.68 (d, 1.8)	113.6
3	-	146.85	-	146.1	3′	-	146.08	-	144.7
4	-	149.50	-	148.4	4′	-	144.93	-	144.7
5	6.77 (dd, 8.0, 2.0)	116.60	6.66 (d, 8.1)	118.0	5′	6.68 (d, 8.0)	116.34	7.14 (d, 8.4)	116.2
6	6.91 (dd, 2.0, 8.0)	123.04	6.74 (d, 8.1)	121.2	6′	6.63 (dd, 8.0, 2.0)	121.89	6.82 (dd, 8.4, 1.8)	117.8
7	7.51 (d, 15.5)	146.79	7.70 (d, 16.0)	142.8	7′	3.10 (dd, 14.5, 3.5), 2.94 (dd, 14.5, 10.0)	38.93	5.85 (d, 4.0)	88.0
8	6.27 (d, 15.5)	115.77	6.23 (d, 16.0)	117.8	8′	5.09 (dd, 10.0, 3.5)	77.79	4.31 (d, 4.0)	57.2
9	-	169.24	-	169.4	9′	-	177.64	-	174.0

^a^ Data were from reference [55] and recorded in CD_3_OD. ^b^ Data were from reference [68] and recorded in (CD_3_)_2_CO-D_2_O(10:1).

**Table 14:** ^1^H and ^13^C NMR Data of compound **121** (*δ* in ppm, *J* in Hz).

Position	Verminoside (121)		Position	Verminoside (121)		Position	Verminoside (121)	
	^1^H (600 MHz)	^13^C (150 MHz)		^1^H (600 MHz)	^13^C (150 MHz)		^1^H (600 MHz)	^13^C (150 MHz)
1	-	127.8	1′	4.80	99.8	1″	5.16	95.2
2	7.07	115.3	2′	3.28	75.0	2″	6.37	142.5
3	-	147.0	3′	3.42	77.8	3″	4.98	103.1
4	-	149.9	4′	3.28	71.9	4″	2.60	36.9
5	6.79	116.6	5′	3.30	78.8	5″	5.03	81.4
6	6.97	123.3	6′	3.93, 3.65	63.1	6″	3.70	60.4
7	7.60	147.7				7″	-	67.0
8	6.32	114.6				8″	2.62	43.3
9	-	169.1				9″	4.17, 3.84	61.4

Data were from reference [90] and recorded in CD_3_OD.

## Data Availability

Data are contained within the article and Supplementary Materials.

## Supplementary Materials

The following supporting information can be downloaded at: https://www.mdpi.com/article/10.3390/molecules29071625/s1, Table S1. NMR Spectroscopic Data (δ) of Compounds **1**–**10**. Table S2. NMR Spectroscopic Data (δ) of Compounds **11**–**17**. Table S3. NMR Spectroscopic Data (δ) of Compounds **18**–**25**. Table S4. NMR Spectroscopic Data (δ) of Compounds **26**–**28**. Table S5. NMR Spectroscopic Data (δ) of Compounds **29**–**37**. Table S6. NMR Spectroscopic Data (δ) of Compounds **38**–**49**. Table S7. NMR Spectroscopic Data (δ) of Compounds **50**–**59**. Table S8. NMR Spectroscopic Data (δ) of Compounds **60**–**71**. Table S9. NMR Spectroscopic Data (δ) of Compounds **72**–**81**. Table S10. NMR Spectroscopic Data (δ) of Compounds **82**–**91**. Table S11. NMR Spectroscopic Data (δ) of Compounds **92**–**102**. Table S12. NMR Spectroscopic Data (δ) of Compounds **103**–**113**. Table S13. NMR Spectroscopic Data (δ) of Compounds **114**–**124**. Table S14. NMR Spectroscopic Data (δ) of Compounds **125**–**135**. Table S15. NMR Spectroscopic Data (δ) of Compounds **136**~**146**. Table S16. NMR Spectroscopic Data (δ) of Compounds **147**–**154**. Table S17. NMR Spectroscopic Data (δ) of Compounds **155**–**162**. Table S18. NMR Spectroscopic Data (δ) of Compounds **163**–**173**.
