# Supplementary material for: Plant-Derived Caffeic Acid and Its Derivatives: An Overview of Their NMR Data and Biosynthetic Pathways
Source: Molecules. 2024 Apr 4;29(7):1625. doi: 10.3390/molecules29071625 (PMC11013677; doi:10.3390/molecules29071625)
Supplement: Supplementary file 1 [file molecules-29-01625-s001.zip › molecules-2909264-supplementary.pdf]

Supporting Information for:

Caffeic acid and its derivatives: An overview of their NMR data and biosynthetic pathways

Jiahui Yu [1,†], Jingchen Xie [1,†], Miao Sun [1], Suhui Xiong [1], Chunfang Xu [1], Zhimin Zhang [1], Minjie Li [1], Chun Li [2], Limei Lin [1,*]

1 Key Laboratory for Quality Evaluation of Bulk Herbs of Human Province, School of Pharmacy, Human University of Chinese Medicine, Changsha, 410208, PR China

2 Institute of Chinese Materia Medica, China Academy of Chinese Medical Sciences, Beijing, 100700, PR China

† These authors contributed equally to this paper.

* Correspondence: limei_lin@hnucm.edu.cn.

Table of contents

Table S1. NMR Spectroscopic Data (δ) of Compounds 1-10 3

Table S2. NMR Spectroscopic Data (δ) of Compounds 11-17 5

Table S3. NMR Spectroscopic Data (δ) of Compounds 18-25 7

Table S4. NMR Spectroscopic Data (δ) of Compounds 26~28 9

Table S5. NMR Spectroscopic Data (δ) of Compounds 29~37 11

Table S6. NMR Spectroscopic Data (δ) of Compounds 38~49 13

Table S7. NMR Spectroscopic Data (δ) of Compounds 50~59 16

Table S8. NMR Spectroscopic Data (δ) of Compounds 60~71 19

Table S9. NMR Spectroscopic Data (δ) of Compounds 72~81 22

Table S10. NMR Spectroscopic Data (δ) of Compounds 82~91 25

Table S11. NMR Spectroscopic Data (δ) of Compounds 92~102 28

Table S12. NMR Spectroscopic Data (δ) of Compounds 103~113 31

Table S13. NMR Spectroscopic Data (δ) of Compounds 114~124 34

Table S14. NMR Spectroscopic Data (δ) of Compounds 125~135 37

Table S15. NMR Spectroscopic Data (δ) of Compounds 136~146 41

Table S16. NMR Spectroscopic Data (δ) of Compounds 147~154 44

Table S17. NMR Spectroscopic Data (δ) of Compounds 155~162 47

Table S18. NMR Spectroscopic Data (δ) of Compounds 163~173 50

Table S1. NMR Spectroscopic Data (δ) of Compounds 1-10

No.	1	2	3	4	5	6	7	8	9	10
Caffeoyl										
C_1/H_1	127.7/-	127.7/-	127.9/-	127.8/-	125.5/-	127.8/-	126.9/-	127.5/-	127.8/-	127.8/-
C_2/H_2	115.1/7.04	115.1/7.03	116.6/7.03	115.2/7.03	114.7/7.02	116.2/7.17	115.1/7.03	115.0/7.20	114.6/7.09	115.3/7.03
C_3/H_3	146.8/-	146.8/-	146.9/-	149.8/-	148.3/-	145.5/-	144.6/-	145.3/-	144.0/-	146.8/-
C_4/H_4	149.4/-	149.5/-	149.7/-	146.9/-	145.5/-	148.6/-	147.1/-	148.5/-	146.5/-	149.6/-
C_5/H_5	116.5/6.75	116.5/6.76	123.0/6.77	116.5/6.93	121.3/6.74	115.0/7.04	113.8/6.92	115.6/6.91	115.7/6.87	116.5/6.77
C_6/H_6	122.8/6.93	122.9/6.94	115.4/6.94	122.9/6.77	123.0/6.96	122.4/6.87	121.9/6.80	122.3/7.09	122.6/7.01	123.1/6.91
C_7/H_7	147.1/7.57	146.9/7.52	147.0/7.53	147.0/7.53	144.9/7.40	146.2/7.54	145.0/7.52	146.1/6.32	144.9/7.57	147.0/7.50
C_8/H_8	115.5/6.31	114.8/6.24	115.3/6.24	115.4/6.19	115.7/6.14	115.0/6.29	115.0/6.20	116.2/7.58	116.0/6.27	115.3/6.21
C_9/H_9	171.1/-	169.8/-	169.5/-	171.0/-	165.8/-	167.4/-	167.9/-	167.3/-	168.0/-	169.4/-
$C_{1'}/H_{1'}$		52.0/3.75	61.5/4.22	176.1/-	175.2/-	64.6/4.14	64.6/4.14	64.5/4.18	65.1/4.19	131.0/-
$C_{2'}/H_{2'}$			14.8/1.30	29.8/2.56	22.8/1.98	32.5/1.68	31.8/1.66		32.1/1.68	117.2/6.71
$C_{3'}/H_{3'}$						29.92/1.34		23.2-32.5/1.32-1.72	22-32	146.3/-
$C_{4'}/H_{4'}$						29.89/1.34	22.6-29.9/1.22-1.37		/	144.9/-
$C_{5'}/H_{5'}$						29.5/1.34			1.10-1.40	116.6/6.70

$C_{6'}/H_{6'}$						26.6/1.34				121.4/6.56
$C_{7'}/H_{7'}$						23.2/1.34				35.7/2.80
$C_{8'}/H_{8'}$						14.3/0.89				66.6/4.27
$C_{9'}/H_{9'}$										
~										
$C_{17'}/H_{17'}$										
$C_{18'}/H_{18'}$							14.0/0.85			
$C_{19'}/H_{19'}$										
$C_{20'}/H_{20'}$								14.2/0.92		
$C_{21'}/H_{21'}$										
$C_{22'}/H_{22'}$									14.3/0.88	
Determination condition	100MHz,<br>CD_3OD<br>/<br>400MHz,<br>CD_3OD	125MHz,<br>CD_3OD /<br>500MHz,<br>CD_3OD	100MHz,<br>CD_3OD<br>/<br>400MHz,<br>CD_3OD	125MHz,<br>CD_3OD<br>/<br>500MHz,<br>CD_3OD	150MHz,<br>DMSO-d_6<br>/<br>600MHz, DMSO-d_6	75MHz<br>acetone-d_6,<br>/<br>300MHz<br>acetone-d_6	100MHz,<br>$CDCl_3$-CD_3OD<br>/<br>400MHz,<br>$CDCl_3$-CD_3OD	acetone-d_6,<br>/<br>acetone-d_6	100MHz,<br>$CDCl_3$<br>/<br>400MHz,<br>$CDCl_3$	150MHz,<br>MeOH-d_4<br>/<br>600MHz,<br>MeOH-d_4
Reference	[12]	[13]	[14]	[15]	[16]	[17]	[18]	[19]	[20]	[21]

Table S2. NMR Spectroscopic Data (*δ*) of Compounds 11-17

No.	11	12	13	14	15	16	17
Caffeoyl							
C_1/H_1	128.04/-	125.23/-	126.19,126.19/-	128.69,127.56/	127.76,128.31/-	127.67,128.57/-	127.64/-
C_2/H_2	117.43/7.10	115.25/7.14-7.05	113.65,113.42/7.19,7.09	113.90,112.42/7.16,7.13	116.44,131.90/7.07,7.49	116.05,114.08/7.12,7.05	114.11/7.09
C_3/H_3	145.49/-	148.87/-	146.12,147.13/-	151.73,148.00/	145.43,117.11/-	147.75,148.87/-	148.93/-
C_4/H_4	148.64/-	145.61/-	146.47,149.45/-	149.90,148.27/	148.10,148.32/-	150.79,145.44/-	145.45/-
C_5/H_5	114.26/6.87	115.81/6.79	115.30,110.63/7.02,6.83	116.48,115.06/7.02,7.00	115.07,117.11/6.77,6.81	117.45,133.61/6.77	133.61/-
C_6/H_6	116.49/7.03,7.05	121.74/7.14-7.05	122.71,122.06/7.10,6.82	123.37,123.17/6.83,6.83	117.21,131.90/6.9,7.49	124.27,123.70/7.01,7.02	123.76/7.01
C_7/H_7	148.90/7.62	147.05/7.56	143.33,143.33/7.65,7.59	147.87,146.84/7.72,7.69	147.91,147.91/7.60,7.70	149.29,149.20/7.55,7.53	149.47/7.54
C_8/H_8	124.33/6.33	112.36/6.37	114.87,114.92/6.40,6.35	114.96,114.70/6.48,6.43	115.33,124.12/6.41,6.45	114.85,114.90/6.36,6.33	114.70/6.34
C_9/H_9	169.40/-	165.53/-	167.64,167.64/-	167.89,167.71/	169.83/-	168.71,168.75/-	168.62/-
$C_{1'}/H_{1'}$	172.46/-	167.48/-	172.25/-	170.60/	173.15/-	171.15/-	170.75/-
$C_{2'}/H_{2'}$	71.54/5.60	70.69/5.69	73.34/5.49	73.12/5.83	72.83/5.71	73.87/5.70	73.56/5.66
$C_{3'}/H_{3'}$	75.25/4.91	70.69/5.69	73.34/5.49	73.12/5.83	72.83/5.71	73.87/5.70	73.56/-
$C_{4'}/H_{4'}$	175.08/-	167.48/-	172.25/-	170.60/	173.15/-	171.15/-	170.75/-
$C_{1''}/H_{1''}$			55.83/3.78	56.33/3.93		128.55/-	128.66/-

$C_{2''}/H_{2''}$						118.11/6.57	118.12/6.58
$C_{3''}/H_{3''}$						147.57/-	147.56/-
$C_{4''}/H_{4''}$						146.78/-	146.78/-
$C_{5''}/H_{5''}$						117.03/6.56	117.05/6.52
$C_{6''}/H_{6''}$						122.30/6.42	122.31/6.42
$C_{7''}/H_{7''}$						39.74/3.07,2.79	39.75/3.08,2.79
$C_{8''}/H_{8''}$						49.12/4.11	49.20/4.11
$C_{9''}/H_{9''}$						178.55/-	178.55/-
Determination condition	75.5MHz, D_2O / 300MHz, D_2O	500MHz, DMSO-d_6 / 125MHz, DMSO-d_6	D_2O / D_2O	CD_3OD / CD_3OD	75.5MHz, D_2O / 300MHz, D_2O, CD_3OD	DMSO-d_6 / 400MHz, CD_3OD	DMSO-d_6 / 400MHz, CD_3OD
Reference	[22]	[23]	[24]	[24]	[22]	[25]	[25]

Table S3. NMR Spectroscopic Data (δ) of Compounds 18-25

No.	18	19	20	21	22	23	24	25
caffeoyl C_1/H_1	129.7/-	126.5/-	126.4/-	126.5/-	126.5/-	128.3/-	128.2/-	128.5/-
C_2/H_2	121.2/6.69	114.1/6.95	114.1/6.95	114.1/6.94	114.1/6.94	117.8/7.08	115.2/7.02	115.0/7.02
C_3/H_3	145.0/-	145.7/-	145.7/-	145.7/-	145.7/-	145.8/-	146.7/-	146.6/-
C_4/H_4	146.0/-	147.7/-	147.6/-	147.6/-	147.6/-	147.4/-	148.9/-	148.6/-
C_5/H_5	117.4/6.67	116.0/6.75	115.9/6.75	116.0/6.74	115.9/6.74	116.0/6.71	116.5/6.78	116.5/6.77
C_6/H_6	121.7/6.56	120.8/6.85	120.6/6.85	120.6/6.82	120.7/6.82	123.4/6.85	122.2/6.91	122.2/6.89
C_7/H_7	142.3/6.65	140.1/7.25	140.0/7.25	139.7/7.19	139.8/7.20	139.5/6.59	143.0/7.40	141.9/7.34
C_8/H_8	116.4/7.49	118.2/6.44	118.1/6.41	118.4/6.42	118.2/6.41	120.6/5.80	117.7/6.43	118.9/6.41
C_9/H_9	168.3/-	165.4/-	165.6/-	165.4/-	165.5/-	169.8/-	169.0/-	168.3/-
$C_{1'}/H_{1'}$	135.1/-	172.8/-	173.5/-	128.7/-	127.9/-	129.3/-	129.5/-	131.0/-
$C_{2'}/H_{2'}$	128.7/7.53	48.9/4.63	51.4/4.33	116.7/6.62	130.2/7.03	117.1/6.64	113.8/6.80	117.7/6.72
$C_{3'}/H_{3'}$	129.7/7.33-7.38	36.7/2.62,2.73	26.7/1.82,2.01	145.1/-	115.2/6.65	146.2/-	148.9/-	145.8/-
$C_{4'}/H_{4'}$	130.7/7.33-7.38	172.0/-	30.2/2.30	144.0/-	156.1/-	145.2/-	146.5/-	144.8/-
$C_{5'}/H_{5'}$	129.7/7.33-7.38		173.2/-	115.5/6.60	115.2/6.65	116.3/6.68	116.2/6.73	116.1/6.67
$C_{6'}/H_{6'}$	128.7/7.53			120.0/6.47	130.2/7.03	121.6/6.46	122.8/6.66	122.1/6.58

$C_{7'}/H_{7'}$	37.8/2.91,3.10			35.0/2.72,2.91	36.4/2.78,2.98	37.8/2.87,2.96	38.2/2.97,3.12	38.7/2.94,3.12
$C_{8'}/H_{8'}$	55.5/4.72			54.3/4.42	54.2/4.48	55.4/4.69	55.7/4.76	57.5/4.61
$C_{9'}/H_{9'}$	175.0/-			173.7/-	173.5/-	173.6/-	173.8/-	178.2/-
$C_{10'}/H_{10'}$						52.6/3.69	56.4/3.83	
$C_{11'}/H_{11'}$							52.7/3.72	
Determination condition	100.5 MHz, MeOH-d_4<br>/<br>400 MHz, MeOH-d_4	100 MHz, DMSO-d_6<br>/<br>400 MHz, DMSO-d_6	100 MHz, DMSO-d_6<br>/<br>400 MHz, DMSO-d_6	100 MHz, DMSO-d_6<br>/<br>400 MHz, DMSO-d_6	100 MHz, DMSO-d_6<br>/<br>400 MHz, DMSO-d_6	CD_3OD<br>/<br>CD_3OD	CD_3OD<br>/<br>CD_3OD	CD_3OD<br>/<br>CD_3OD
Reference	[26]	[27]	[27]	[27]	[27]	[28]	[28]	[28]

Table S4. NMR Spectroscopic Data (δ) of Compounds 26~28

No.	26	27	28
Shikimic C_1/H_1	128.5/-	131.0/-	132.8/-
C_2/H_2	138.6/6.70	138.2/6.84	132.7/6.48
C_3/H_3	65.5/4.30	65.6/4.61	70.9/5.53
C_4/H_4	68.3/3.78	75.0/5.02	69.9/3.79
C_5/H_5	70.5/4.48	65.8/4.20	68.3/3.87
C_6/H_6	27.5/2.67, 2.19	32.1/2.30, 2.72	31.9/2.52, 2.12
C_7/H_7	168.0/-	169.8/-	169.7/-
Caffeoyl $C_{1'}/H_{1'}$	125.9/-	127.8/-	126.8/-
$C_{2'}/H_{2'}$	114.7/7.00	115.1/7.04	116.2/7.06
$C_{3'}/H_{3'}$	145.2/-	147.2/-	147.1/-
$C_{4'}/H_{4'}$	149.7/-	146.7/-	150.1/-
$C_{5'}/H_{5'}$	116.4/6.26	116.5/6.77	117.3/6.78
$C_{6'}/H_{6'}$	121.6/6.78	123.0/6.95	122.6/7.00
$C_{7'}/H_{7'}$	149.5/7.06	147.2/7.59	146.6/7.52
$C_{8'}/H_{8'}$	114.9/5.12	115.2/6.33	115.7/6.28

$C_{9'}/H_{9'}$	166.7/-	168.9/-	167.2/-
Determination condition	125.77 MHz, DMSO-d_6 / 500.13 MHz, DMSO-d_6	75MHz，CD_3OD / 300MHz，CD_3OD	125MHz，CD_3OD / 500MHz，DMSO-d_6
Reference	[29]	[30]	[31]

Table S5. NMR Spectroscopic Data (δ) of Compounds 29~37

No.	29	30	31	32	33	34	35	36	37
Quinic acid C_1/H_1	75.4/-	75.8/-	75.91/-	76.2/-	76.6/-	75.0/-	75.5/-	73.9/-	81.6/-
C_2/H_2	36.7/2.20,2.13	38.0/1.99,2.19	38.70/2.12	37.5/2.20	38.4/ 2.06,2.17	40.7/2.01-2.28	41.5/1.9-2.2	39.4/2.00-2.24	35.9/3.52,2.57
C_3/H_3	73.0/5.34	70.3/4.13	73.21/5.36	71.8/5.30	69.6/4.28	64.3/4.29	68.5/4.140	67.2/4.13	67.9/4.17
C_4/H_4	74.8/ 3.63	72.5/3.72	70.91/3.76	72.3/3.77	79.3/4.79	77.1/4.84	74.8/3.643	72.4/3.70	76.2/4.09
C_5/H_5	68.3/4.14	72.1/5.26	71.49/4.19	70.1/4.17	65.5/4.27	67.6/4.29	73.1/5.353	71.2/5.38	69.5/2.45
C_6/H_6	41.5/1.95, 2.13	37.7/2.18,2.18	37.92/2.12	37.8/2.02,2.25	42.7/2.00,2.20	37.0/2.01-2.28	36.8/-	34.9/2.00-2.24	39.6/2.20,1.96
C_7/H_7	178.3/-	175.4/-	173.99/-	175.5/-	177.3/-	174.3/-	178.6/-	175.0/-	175.4/-
C_8/H_8		53.0/3.68	61.76/4.15	66.3/4.11		51.5/3.77		51.4/3.75	
C_9/H_9			14.34/1.23	31.8/1.64					
C_{10}/H_{10}				20.1/1.38					
C_{11}/H_{11}				14.2/0.92					
Caffeoyl $C_{1'}/H_{1'}$	127.9/-	127.6/-	127.57/-	127.9/-	127.8/-	126.5/-	122.9/6.940	126.5/-	127.8/-
$C_{2'}/H_{2'}$	115.1/7.04	115.1/7.03	116.39/7.17	115.0/7.06	115.1/7.06	113.7/7.08	115.9/6.305	115.0/7.06	115.1/7.05
$C_{3'}/H_{3'}$	146.79/-	146.8/-	115.73/-	150.1/-	146.8/-	145.4/-	146.9/7.583	145.4/-	146.8/-
$C_{4'}/H_{4'}$	149.4/-	149.7/-	148.78/-	147.3/-	149.6/-	148.2/-	128.1/-	148.1/-	149.6/-

$C_{5'}/H_{5'}$	116.4/6.76	116.5/6.77	146.29/7.04	116.2/6.81	116.5/6.77	115.1/6.80	115.2/7.041	114.3/6.89	116.5/6.79
$C_{6'}/H_{6'}$	122.9/ 6.93	123.0/6.94	115.13/6.88	122.7/6.98	123.0/ 6.96	121.5/6.99	145.0/-	121.5/6.97	123.0/6.96
$C_{7'}/H_{7'}$	146.80/7.58	147.2/7.51	145.85/7.55	147.0/7.55	147.1/7.65	145.7/7.66	149.8/-	145.4/7.61	147.3/7.56
$C_{8'}/H_{8'}$	115.8/6.30	115.0/6.21	122.54/6.26,6.21	114.8/6.24	115.4/6.37	113.9/6.38	116.5/6.770	113.7/6.33	115.3/6.28
$C_{9'}/H_{9'}$	169.0/-	168.3/-	167.98/-	168.7/-	169.0/-	167.5/-	169.1/-	167.5/-	168.1/-
Determination condition	125MHz, CD_3OD / 500MHz, CD_3OD	100MHz, CD_3OD / 400MHz, CD_3OD	50.7MHz, acetone-D_6 / 200MHz, acetone-D_6	150MHz, CD_3OD / 600MHz, CD_3OD	125MHz, CD_3OD / 500MHz, CD_3OD	125MHz, CD_3OD / 500MHz, CD_3OD	125.8MHz, CD_3OD / 500.2MHz, CD_3OD	125MHz, CD_3OD / 500MHz, CD_3OD	75.4MHz, MeOH-D_4 / 300.1MHz, MeOH-D_4
Reference	[32]	[33]	[34]	[35]	[32]	[36]	[37]	[36]	[38]

Table S6. NMR Spectroscopic Data (*δ*) of Compounds 38~49

No.	38	39	40	41	42	43	44	45	46	47	48	49
Quinic acid C_1/H_1	74.8/-	74.6/-	74.27/-	76.1/-	75.8/-	76.6/-	76.33/-	82.7/-	76.8/-	80.0/-	79.5/-	72.7/-
C_2/H_2	37.7/2.1-2.3	36.7/2.31,2.19	36.95/2.26	39.4/2.0-2.4	38.6/2.26,2.26	42.0/2.0-2.4	42.12/2.15	35.5/2.25, 2.69	40.2/2.15,1.87	34.2/2.49,2.16	34.3/2.88,2.45	35.5/2.30,2.00
C_3/H_3	72.2/5.400	72.0/5.31	71.59/5.40	69.0/5.625	68.6/5.56	65.9/4.373	66.80/4.32	70.5/5.45	68.4/5.55	69.0/4.00	71.0/5.37	68.5/5.46
C_4/H_4	70.8/3.981	69.7/3.98	69.67/4.05	75.8/5.121	74.9/5.12	75.3/4.993	70.63/5.04	73.2/3.72	76.1/4.98	72.5/3.50	70.7/3.99	68.6/5.21
C_5/H_5	72.6/5.437	72.2/5.40	71.83/5.40	69.4/4.372	69.1/4.36	70.2/5.635	75.94/5.62	69.6/4.22	69.1/4.24	70.6/5.24	70.2/5.44	67.8/5.46
C_6/H_6	36.1/2.1-2.3	35.7/2.34,2.15	35.64/2.26	38.4/2.0-2.4	38.4/2.32,2.10	37.1/2.0-2.4	37.57/2.35,2.12	37.1/1.98, 2.69	38.3/2.15	37.9/2.33,1.78	35.3/2.55,2.10	35.5/2.21,2.07
C_7/H_7	177.4/-	175.6/-	174.43/-	176. 8/-	175.2/-	178.0/-	176.91/-	176.8/-	177.6/-	176.9/-	173.5/-	175.3/-
C_8/H_8		53.0/3.70	61.72/4.13		53.1/3.72		37.57/3.76					
C_9/H_9			14.30/1.22									
Caffeoyl $C_{1'}/H_{1'}$	128.0,127.9 /-	127.9,127.6/-	127.70,127.63/-	127.8,127.7/-	127.7,127.5/-	127.9,127.8/-	128.52,128.46/-	126.8,126.5/-	125.8, 126.1/-	124.8, 125.4/-	126.4,126.3/7.07,6.96,6.84	125.3,125.4,125.5/-

$C_{2'}/H_{2'}$	115.4,115.3/7.067,7.067	115.2,114.9/7.07,7.06	116.43,116.37/7.18,7.16	115.3,115.2/7.024,7.005	115.2,115.2/7.04,7.02	115.3,115.2/7.038,7.016	115.95/7.03,7.02	113.7,113.6/7.05	115.4, 115.4/7.02,7.02	114.3, 114.3/7.06,7.00	113.7,113.6/-	114.9,114.9,114.9/7.04,7.03,7.01
$C_{3'}/H_{3'}$	147.3,147.1/-	146.9,146.8/-	116.14,115.52/-	146.8,146.8/-	146.8,146.8/-	147.43,147.37/-	147.55/-	145.3,144.8/-	146.2,146.2/-	145.8,145.8/-	145.6,145.4/-	145.6,145.6,145.6/-
$C_{4'}/H_{4'}$	149.6,149.5/-	149.7,149.5/-	148.89,148.70/-	149.7,149.7/-	149.8,149.7/-	149.7,149.7/-	150.38/-	148.2,147.8/-	149.2,149.2/-	149.3,148.4/-	146.2,146.0/-	148.4,148.5,148.6/-
$C_{5'}/H_{5'}$	116.6,116.6/6.790,6.788	116.6,116.5/6.79,6.78	146.32,146.28/7.05,6.94	116.5,116.5/6.750,6.745	116.5,116.5/6.76,6.76	116.6,116.5/6.869	117.26/6.76,6.73	115.1,115.0/6.77	116.4, 116.4/6.75,6.75	115.6, 115.6/6.70,6.73	115.2,115.1/6.75,6.65,6.54	115.7,115.8,115.8/6.74,6.76,6.70
$C_{6'}/H_{6'}$	123.1,123.0/6.979,6.963	123.1,123.0/6.97,6.97	115.44,115.12/6.88,6.83	123.2,123.2/6.905	123.1,123.1/6.93,6.91	123.3,123.1/6.924	124.03,123.90/6.91,6.88	121.6,121.4/6.94	122.0, 122.0/6.95,6.95	121.1, 120.4/6.94,6.90	121.8,121.7/6.97,6.83,6.64	121.4,121.5,121.6/6.97,6.99,6.92
$C_{7'}/H_{7'}$	146.8,146.8/7.621,7.579	147.4,147.1/7.62,7.55	122.61,122.48/7.59,7.56	147.8,147.6/7.600,7.518	147.7,147.7/7.61,7.51	146.9,146.8/7.574,7.543	148.19/7.56,7.54	145.5,145.4/7.57	146.1, 146.1/7.50,7.50	144.8, 143.2/7.46,7.34	148.3/7.64,7.56,7.51	146.0,145.7,145.5/7.45,7.49,7.42

$C_{8'}/H_{8'}$	115.7,115.2/6.346,6.265	115.5,115.2/6.34,6.22	146.11,145.66/6.31	114.81,114.76/6.286,6.192	114.7,114.6/6.30,6.18	115.1,115.0/6.252,6.284	115.80,115.60/6.26	115.5,114.2/6.32	114.2, 114.2/6.24,6.24	113.6, 116.1/6.73, 6.12	114.3,114.2/6.34,6.30,6,25	114.1,113.5,113.2/6.17,6.19,6.23
$C_{9'}/H_{9'}$	168.9,168.4/-	168.7,168.0/-	167.00,166.58/-	168.6,168.3 /-	168.5,167.9/-	168.7,168.6/-	169.32,169.23/-	167.8,166.9/-	166.6,166.8/-	166.2,165.6/-	167.3,167.2,166.5/-	166.0,165.7,165.5/-
Determination condition	125.8MHz, CD_3OD / 500.2MHz, CD_3OD	125MHz, CD_3OD / 500MHz, CD_3OD	50.7MHz, acetone-D_6 / 200MHz, acetone-D_6	125.8MHz, CD_3OD / 500.2MHz， CD_3OD	125MHz, CD_3OD / 500MHz, CD_3OD	125.8MHz, CD_3OD / 500.2MHz, CD_3OD	methanol-d_4 / methanol-d_4	125MHz, CD_3OD / 500MHz, CD_3OD	100MHz, DMSO-d_6 / 400MHz， DMSO-d_6	75MHz, DMSO-d_6 / 300MHz, DMSO-d_6	125MHz, CD_3OD / 500MHz, CD_3OD	125MHz, DMSO-d_6 / 500MHz, DMSO-d_6
Reference	[37]	[39]	[34]	[37]	[39]	[37]	[40]	[41]	[42]	[43]	[44]	[45]

Table S7. NMR Spectroscopic Data (δ) of Compounds 50~59

No.	50	51	52	53	54	55	56	57	58	59
Quinic acid C_1/H_1	80.0/-	79.5/-	79.3/-	74.0/-	73.1/-	72.4/-	72.7/-	72.5/-	74.7/-	76.2/-
C_2/H_2	32.3/2.39-2.55	34.7/2.32-2.60	32.6/2.52-2.72,2.52-2.72	37.8/1.97.2.20	40.0/2.14,1.99	36.4/2.19,2.00	35.5/2.10,2.04	35.5/2.25,1.97	35.7/2.10-2.34	38.8/2.03,2.24
C_3/H_3	70.3/5.25	65.6/4.24	69.0/5.62	66.7/4.30	68.9/5.53	71.2/5.31	70.8/5.29	67.8/5.43	72.1/5.38	69.1/4.29
C_4/H_4	71.9/3.84	74.4/4.92	72.2/5.24	75.2/4.97	73.1/4.92	68.3/3.84	67.5/3.84	72.1/5.02	69.8/3.95	75.4/5.06
C_5/H_5	69.6/5.22	67.2/5.46	67.6/5.60	67.4/5.57	65.3/4.16	70.0/5.28	70.6/5.19	64.8/4.05	72.2/5.29	69.5/5.48
C_6/H_6	36.6/1.92,2.39-2.54	36.8/2.02-2.07,2.32-2.60	37.3/2.52-2.72,2.19	37.8/2.08,2.20	36.5/1.99,1.91	34.7/2.00,1.98	34.7/2.02,2.24	39.1/2.12,1.93	36.8/2.10-2.34	39.0/2.23
C_7/H_7	173.7/-	173.8/-	173.6/-	175.0/-	177.4/-	175.3/-	173.9/-	174.0/-	175.6/-	175.6/-
C_8/H_8							51.9/3.60	51.7/3.64	53.0/3.67	53.5/3.65
$C_{1'}/H_{1'}$	172.7/-	172.2/-	172.1,173.2/-	119.6/-	119.7/-	119.8/-	120.0/-	125.4/-	168.6/-	168.8/-
$C_{2'}/H_{2'}$	29.4/2.39-2.54	29.4/2.32-2.60	29.6,29.8/2.52-2.72	107.4/7.21	106.9/7.20	106.8/7.31	107.3/7.29	114.8/7.04	116.3/6.44	116.0/6.33
$C_{3'}/H_{3'}$	29.2/2.39-2.54	29.2/2.32-2.60	29.0,29.1/2.52-2.72	147.8/-	147.4/-	146.9/-	147.4/-	145.6/-	147.2/7.65	148.2/7.56
$C_{4'}/H_{4'}$	172.1/-	172.1/-	172.1,172.5/-	141.1/-	140.5/-	139.9/-	140.5/-	148.5/-	126.8/-	127.0/-

$C_{5'}/H_{5'}$				147.8/-	147.4/-	146.9/-	147.4/-	115.7/6.74	106.9/-	107.3/6.77
$C_{6'}/H_{6'}$				107.4/7.21	106.9/7.20	106.8/7.31	107.3/7.29	121.4/6.98	149.5/-	149.8/-
$C_{7'}/H_{7'}$				165.5/-	164.7/-	164.7/-	165.1/-	145.4/7.48	139.5/-	140.0/-
$C_{8'}/H_{8'}$				56.3/3.79	55.9/3.72	55.5/3.81	56.1/3.81	113.9/6.26	149.5/-	149.8/-
$C_{9'}/H_{9'}$				56.3/3.79	55.9/3.72	55.5/3.81	56.1/3.81	165.9/-	106.9/6.90	107.3/6.77
$C_{10'}/H_{10'}$								56.0/3.76	56.8/3.86	57.2/3.75
$C_{11'}/H_{11'}$								56.0/3.76	56.8/3.86	57.2/3.75
Caffeoyl $C_{1''}/H_{1''}$	125.9,126.0/-	126.0,126.0/-	127.4,127.5/-	125.6/-	125.4/-	125.0/-	125.3/-	124.4/-	168.0/	168.3/-
$C_{2''}/H_{2''}$	115.4,115.4/7.07,7.07	115.3,115.4/7.06,7.06	115.4,115.5/7.15,7.22	115.2/7.00	115.8/7.01	114.3/7.04	114.7/7.03	106.2/6.95	114.8/6.20	114.9/6.09
$C_{3''}/H_{3''}$	146.1,146.1/-	146.1,146.1/-	146.3,146.3/-	145.9/-	145.6/-	145.0/-	145.6/-	148.0/-	147.4/7.52	148.1/7.43
$C_{4''}/H_{4''}$	149.0,149.0/-	148.9,149.0/-	148.9,149.0/-	148.8/-	148.5/-	147.8/-	148.5/-	138.3/-	127.6/-	127.9/-
$C_{5''}/H_{5''}$	116.3,116.4/6.78,6.78	116.3,116.4/6.77,6.79	116.3,116.4/6.86,6.87	116.0/6.74	113.9/6.74	115.2/6.77	115.8/6.77	148.0/-	115.1/7.02	115.6/6.93
$C_{6''}/H_{6''}$	122.6,122.7/7.01,7.01	121.7,121.9/7.01,7.01	122.7,122.8/7.04,7.11	121.7/6.95	121.4/6.93	120.7/6.99	121.3/6.99	106.2/6.95	146.9/-	147.2/-
$C_{7''}/H_{7''}$	145.8,145.8/7.49,7.50	146.1,146.1/7.48,7.48	146.5,147.0/7.56,7.65	145.9/7.43	145.4/7.43	144.4/7.44	145.3/7.42	145.5/7.50	149.8/-	150.2/-
$C_{8''}/H_{8''}$	114.5,114.6/6.25,6.27	114.0,114.8/6.20,6.24	115.0,115.2/6.25,6.42	113.9/6.15	114.0/6.24	113.8/6.18	113.8/6.15	115.0/6.46	116.5/6.75	116.9/6.67
$C_{9''}/H_{9''}$	166.5,165.6/-	165.7,166.2/-	166.3,166.5/-	166.0/-	165.9/-	165.2/-	165.3/-	165.8/-	123.1/6.94	123.5/6.83

Determination condition	100MHz, DMSO-d_6 / 400MHz, DMSO-d_6	100MHz, DMSO-d_6 / 400MHz, DMSO-d_6	126MHz, Acetone-d_6 / 500MHz, Acetone-d_6	125MHz, DMSO-d_6 / 500MHz, DMSO-d_6	125MHz, DMSO-d_6 / 500MHz, DMSO-d_6	125MHz, DMSO-d_6 / 500MHz, DMSO-d_6	100MHz DMSO-d_6 / 400MHz DMSO-d_6	100MHz, DMSO-d_6 / 400MHz, DMSO-d_6	125MHz, DMSO-d_6 / 500MHz, DMSO-d_6	75MHz, CD_3OD / 300MHz, CD_3OD
Reference	[46]	[46]	[47]	[48]	[48]	[48]	[49]	[49]	[50]	[51]

Table S8. NMR Spectroscopic Data (δ) of Compounds 60~71

No.	60	61	62	63	64	65	66	67	68	69	70	71
Quinic acid												
C_1/H_1	73.0/-	72.8/-	73.0/-	72.8/-	72.6/-	73.4/-	73.1/-	73.1/-	72.5/-	72.3/-	73.7/-	75.7/-
C_2/H_2	36.0/2.16,1.98	37.5/1.91,2.18	35.6/2.25,1.97	35.0/2.19,1.97	34.6/2.23,2.02	36.9/2.15,2.04	36.1/2.24,2.01	36.0/2.05,1.97	35.1/2.24,1.98	35.4/2.10,1.92	34.4/2.12,1.94	37.5/2.23,2.11
C_3/H_3	68.7/5.53	65.2/4.26	68.4/5.50	71.3/5.32	70.7/5.28	66.2/4.18	65.2/4.14	68.2/5.47	67.9/5.41	69.9/5.14	66.3/4.15	67.4/4.29
C_4/H_4	73.8/4.96	73.1/4.98	72.8/4.97	68.5/3.85	67.5/3.88	73.0/4.99	72.1/4.99	72.6/5.00	72.5/4.99	67.0/3.80	72.7/4.93	74.8/5.02
C_5/H_5	64.5/4.09	67.4/5.52	64.4/4.10	70.6/5.24	70.6/5.16	67.7/5.34	67.7/5.25	65.0/3.98	64.8/4.03	70.9/5.05	68.1/5.26	67.4/5.46
C_6/H_6	39.6/1.98,1.91	37.5/2.02,2.16	39.4/2.01,1.98	36.0/2.00,2.02	37.0/2.14,2.06	37.5/2.13,1.91	37.5/2.23,1.89	39.4/1.90,1.89	39.4/2.11,1.92	34.4/2.12,1.94	37.9/2.15,1.87	36.9/1.99,2.11
C_7/H_7	176.4/-	175.0/-	174.2/-	175.6/-	173.9/-	174.6/-	173.3/-	176.4/-	174.0/-	175.1/-	175.1/-	175.0/-
C_8/H_8			51.8/3.63		51.9/3.59		51.9/3.59		51.7/3.62			
$C_{1'}/H_{1'}$	121.0/-	121.4/-	120.7/-	121.3/-	121.4/-	127.5/-	127.5/-	127.5/-	127.5/-	48.1/-	48.6/-	167.9/-
$C_{2'}/H_{2'}$	112.9/7.42	112.8/7.42	112.8/7.42	113.1/7.50	113.0/7.51	118.2/7.24	118.2/7.26	118.1/7.21	118.2/7.21	43.9/1.69,1.52	44.4/1.69,1.54	48.7/3.29
$C_{3'}/H_{3'}$	147.2/-	147.2/-	147.2/-	147.1/-	147.2/-	129.9/-	129.9/-	129.8/-	129.9/-	63.9/3.90	64.3/3.90	170.0/-

$C_{4'}/H_{4'}$	151.4/-	151.6/-	151.6/-	151.2/-	151.4/-	150.0/-	150.0/-	149.9/-	149.9/-	45.5/1.86,1.61	46.0/1.86,1.61
$C_{5'}/H_{5'}$	115.0/6.83	115.1/6.83	115.1/6.82	114.9/6.86	115.0/6.86	144.0/-	143.9/-	143.9/-	144.0/-	85.8/-	86.2/-
$C_{6'}/H_{6'}$	123.5/7.44	123.7/7.45	123.6/7.43	123.7/7.53	123.7/7.52	112.1/7.24	112.2/7.26	112.1/7.21	112.1/7.23	81.6/-	82.1/-
$C_{7'}/H_{7'}$	164.8/-	165.1/-	164.8/-	165.2/-	165.1/-	145.4/7.61	145.4/7.63	145.1/7.53	145.1/7.55	135.7/6.48	136.7/6.50
$C_{8'}/H_{8'}$	55.4/3.72	55.5/3.77	55.4/3.71	55.6/3.71	55.6/3.80	114.9/6.51	114.8/6.53	114.8/6.42	115.0/6.44	129.3/7.90	129.8/7.92
$C_{9'}/H_{9'}$						166.1/-	165.9/-	165.9/-	166.0/-	150.2/-	151.8/-
$C_{10'}/H_{10'}$						131.8/-	131.8/-	131.8/-	131.8/-	117.4/5.76	117.0/5.78
$C_{11'}/H_{11'}$						110.4/6.90	110.4/6.90	110.4/6.90	110.4/6.90	164.7/-	165.0/-
$C_{12'}/H_{12'}$						147.6/-	147.5/-	147.6/-	147.6/-	75.4/3.62,3.54	75.8/3.60,3.52
$C_{13'}/H_{13'}$						146.5/-	146.5/-	146.5/-	146.6/-	20.0/0.98	20.0/1.01
$C_{14'}/H_{14'}$						115.3/6.74	115.2/6.75	115.1/6.74	115.3/6.74	16.1/0.77	16.6/0.80
$C_{15'}/H_{15'}$						118.7/6.73	118.7/6.73	118.7/6.73	118.7/6.70	20.7/2.05	21.2/2.05
$C_{16'}/H_{16'}$						87.9/5.52	87.9/5.52	87.9/5.52	88.0/5.52		
$C_{17'}/H_{17'}$						52.5/3.48	52.5/3.49	52.5/3.49	52.5/3.47		
$C_{18'}/H_{18'}$						62.6/3.72,3.6	62.6/3.72,3.6	62.5/3.71,3.6	62.5/3.72,3.6		

						5	5	5	9			
$C_{19'}/H_{19'}$						55.6/3.73	55.6/3.73	55.6/3.73	55.6/3.72			
$C_{20'}/H_{20'}$						55.8/3.80	55.8/3.81	55.7/3.78	55.7/3.77			
Caffeoyl $C_{1''}/H_{1''}$	125.4/-	125.3/-	125.1/-	125.5/-	125.0/-	125.4/-	125.0/-	125.4/-	124.9/-	125.6/-	125.9/-	126.4/-
$C_{2''}/H_{2''}$	114.7/6.99	114.8/7.03	114.6/6.98	114.8/7.04	114.5/7.02	114.8/7.01	114.6/7.01	114.8/7.03	115.0/7.02	114.8/7.05	115.3/7.05	113.9/6.98
$C_{3''}/H_{3''}$	145.5/-	145.5/-	145.7/-	145.0/-	145.4/-	145.5/-	145.7/-	145.6/-	145.7/-	145.5/-	146.0/-	145.4/-
$C_{4''}/H_{4''}$	148.4/-	148.5/-	148.9/-	148.4/-	148.9/-	148.5/-	148.9/-	148.4/-	148.0/-	148.4/-	148.9/-	148.3/-
$C_{5''}/H_{5''}$	115.7/6.73	115.7/6.73	115.7/6.72	115.7/6.77	115.8/6.75	115.3/6.74	115.3/6.76	115.3/6.73	115.8/6.74	115.4/6.76	116.1/6.75	115.1/6.71
$C_{6''}/H_{6''}$	121.3/6.91	121.4/6.93	121.4/6.90	121.4/6.99	121.4/6.98	121.4/6.96	121.4/6.96	121.4/6.98	121.7/6.95	121.5/6.98	121.9/6.98	121.7/6.90
$C_{7''}/H_{7''}$	145.2/7.42	145.5/7.42	145.4/7.41	145.6/7.45	145.8/7.42	145.5/7.42	145.7/7.41	145.3/7.47	145.2/7.74	145.1/7.45	146.0/7.44	146.1/7.47
$C_{8''}/H_{8''}$	113.9/6.21	113.6/6.13	113.6/6.20	114.2/6.18	113.4/6.13	113.6/6.15	113.1/6.12	113.9/6.26	113.3/6.23	114.1/6.13	114.0/6.15	113.4/6.15
$C_{9''}/H_{9''}$	165.9/-	165.7/-	165.9/-	165.8/-	165.4/-	165.5/-	165.2/-	165.8/-	165.8/-	165.4/-	165.9/-	166.4/-
Determination condition	75MHz, CD_3OD/ 300MHz, CD_3OD	125MHz, DMSO-d_6/ 500MHz, DMSO-d_6	125MHz, DMSO-d_6/ 500MHz, DMSO-d_6	125MHz, DMSO-d_6/ 500MHz, DMSO-d_6	125MHz, DMSO-d_6/ 500MHz, DMSO-d_6	125MHz, DMSO-d_6/ 500MHz, DMSO-d_6	125MHz, DMSO-d_6/ 500MHz, DMSO-d_6	125MHz, DMSO-d_6/ 500MHz, DMSO-d_6	125MHz, DMSO-d_6/ 500MHz, DMSO-d_6	125MHz, DMSO-d_6/ 500MHz, DMSO-d_6	125MHz, DMSO-d_6/ 500MHz, DMSO-d_6	125MHz, DMSO-d_6/ 500MHz, DMSO-d_6
Reference	[51]	[50]	[50]	[50]	[50]	[50]	[50]	[50]	[50]	[50]	[52]	[52]

Table S9. NMR Spectroscopic Data (δ) of Compounds 72~81

No.	72	73	74	75	76	77	78	79	80	81
Caffeoyl										
$C_{1'}/H_{1'}$		129.3/-	128.12/-	127.67/-	127.70/-	125.4/-	128.3/-	127.2/-	125.94/-	126.0/-
$C_{2'}/H_{2'}$		127.0/-	115.27/7.03	115.34/7.04	115.36/7.05	115.8/7.05	118.5/7.55	123.8/-	130.88/-	128.5/-
$C_{3'}/H_{3'}$		144.1/-	146.85/-	147.03/-	147.03/-	145.6/-	147.6/-	145.5/-	146.06/-	144.4/-
$C_{4'}/H_{4'}$		145.9/-	149.50/-	150.10/-	150.07/-	148.6/-	151.6/-	145.0/-	147.42/-	148.3/-
$C_{5'}/H_{5'}$		113.3/6.72	116.60/6.77	116.66/6.78	116.66/6.78	116.9/6.78	117.9/6.88	115.1/6.75	115.95/6.83	114.8/6.70
$C_{6'}/H_{6'}$		119.0/7.09-6.93	123.04/6.91	123.38/6.95	123.37/6.96	121.5/7.02	126.7/7.20	119.8/7.14	121.72/7.21	120.1/7.06
$C_{7'}/H_{7'}$		146.1/7.78	146.79/7.51	148.14/7.55	148.09/7.56	146.4/7.46	147.5/7.62	148.7/7.89	145.79/8.06	147.6/8.01
$C_{8'}/H_{8'}$		124.4/6.17	115.77/6.27	114.23/6.26	114.29/6.27	113.5/6.24	115.6/6.39	116.7/6.28	116.10/6.33	115.2/6.23
$C_{9'}/H_{9'}$		168.4/-	169.24/-	168.50/-	168.54/-	165.9/-	168.7/-	168.5/-	166.85/-	168.5/-
$C_{10'}/H_{10'}$								32.2/3.84		
$C_{11'}/H_{11'}$								175.7/-		
Dan Shen Su										
C_1/H_1	129.1/-		131.29/-	128.89/-	128.87/-	131.7/-	129.7/-	129.6/-	129.14/-	128.7/-
C_2/H_2	116.9/6.62		117.63/6.77	117.67/6.70	117.75/6.72	114.9/6.76	118.1/6.78	117.6/6.74	117.14/6.82	117.3/6.60

C_3/H_3	144.7/-		146.08/-	146.37/-	146.37/-	145.6/-	146.5/-	146.3/-	144.73/-	146.2/-
C_4/H_4	143.6/-		144.93/-	145.55/-	145.56/-	144.1/-	145.7/-	145.4/-	143.94/-	145.3/-
C_5/H_5	115.2/6.60		116.34/6.68	116.45/6.69	116.44/6.70	116.5/6.97	116.8/6.72	116.4/6.70	114.76/6.71	116.3/6.58
C_6/H_6	120.1/6.45		121.89/6.63	121.92/6.57	121.99/6.58	120.1/6.66	122.3/6.63	122.0/6.62	120.32/6.62	122.0/6.44
C_7/H_7	39.6/2.77,2.58		38.93/2.94,3.10	38.05/3.06,3.00	38.07/3.31,3.03	36.3/3.06,2.94	38.3/3.03,3.11	38.1/3.09,2.99	37.49/3.10,2.99	37.9/2.94,2.91
C_8/H_8	71.7/4.01		77.79/5.09	74.82/5.19	74.95/5.15	73.1/5.06	75.1/5.20	75.3/5.14	73.74/5.21	74.5/5.12
C_9/H_9	175.6/-		177.64/-	172.34/-	171.91/-	171.1/-	173.8/-	173.4/-	171.11/-	172.2/-
C_{10}/H_{10}				52.82/3.70	62.57/4.15					52.7/3.63
C_{11}/H_{11}					14.52/1.21					
$C_{1''}/H_{1''}$		135.9/-				102.4/4.63	104.6/4.85		127.57/-	131.3/-
$C_{2''}/H_{2''}$		116.2/7.09-6.93				73.3/-	75.3/3.54		119.76/7.13	114.0/6.98
$C_{3''}/H_{3''}$		147.1/-				77.1/-	78.9/3.54		145.66/-	146.5/-
$C_{4''}/H_{4''}$		163.5/-				69.8/-	71.9/3.42		146.38/-	146.8/-
$C_{5''}/H_{5''}$		116.7/6.72				75.8/-	78.0/3.54		113.71/6.85	116.4/6.69
$C_{6''}/H_{6''}$		119.5/6.72				60.7/-	63.0/3.75,3.99		120.24/6.93	120.4/6.81
$C_{7''}/H_{7''}$		146.1/6.46							137.44/7.16	137.9/6.57
$C_{8''}/H_{8''}$		124.4/7.09-6.93							116.24/6.72	120.7/7.08

Determination condition	150MHz, DMSO-d_6 / 600MHz, DMSO-d_6	101MHz, $CDCl_3$ / 400MHz, $CDCl_3$	125MHz, CD_3OD / 500MHz, CD_3OD	125MHz, CD_3OD / 500MHz, CD_3OD	125MHz, CD_3OD / 500MHz, CD_3OD	100MHz, DMSO-d_6 / 400MHz, DMSO-d_6	125MHz, CD_3OD / 500MHz, CD_3OD	125MHz, CD_3OD / 500MHz, CD_3OD	DMSO-d_6 / DMSO-d_6	125MHz, CD_3OD / 500MHz, CD_3OD
Reference	[53]	[54]	[55]	[55]	[55]	[56]	[57]	[58]	[59]	[60]

Table S10. NMR Spectroscopic Data (δ) of Compounds 82~91

No.	82	83	84	85	86	87	88	89	90	91
Caffeoyl										
$C_{1'}/H_{1'}$	123.8/-	126.5/-	125.6/-	132.5/-	123.4/-	125.80/-	124.7/-	122.4/-	125.9/-	124.3/-
$C_{2'}/H_{2'}$	129.8/-	132.9/-	131.7/-	124.8/6.77	123.9/7.47	120.55/-	127.6/-	126.8/-	127.3/6.70	127.4/-
$C_{3'}/H_{3'}$	148.3/-	144.5/-	144.5/-	143.9/-	116.2/6.89	144.31/-	148.9/-	147.1/-	148.6/-	146.1/-
$C_{4'}/H_{4'}$	145.0/-	148.2/-	146.4/-	152.2/-	150.8/-	149.72/-	145.2/-	145.3/-	146.7/-	148.4/-
$C_{5'}/H_{5'}$	117.1/6.72	115.3/6.88	113.0/6.89	123.3/-	144.8/-	115.66/6.83	118.3/6.82	116.8/6.63	117.2/6.73	118.0/6.66
$C_{6'}/H_{6'}$	123.0/7.06	119.9/7.38	118.4/7.42	132.8/6.78	131.1/-	123.73/6.83	122.0/7.21	120.6/6.51	121.6/6.74	121.2/6.74
$C_{7'}/H_{7'}$	145.2/7.62	146.9/7.92	145.5/7.91	143.7/7.88	142.5/7.82	139.73/7.54	144.1/7.83	143.1/-	143.9/3.33,3.03	142.8/7.70
$C_{8'}/H_{8'}$	115.9/6.25	116.5/6.47	115.4/6.48	117.4/6.22	116.0/6.34	127.00/-	116.5/6.34	115.6/-	116.2/5.90	117.8/6.23
$C_{9'}/H_{9'}$	168.3/-	174.5/-	170.8/-	168.1/-	166.7/-	168.88/-	168.2/	166.1/-	167.9/-	169.4/-
Dan Shen Su										
C_1/H_1	129.2/-	129.9/-	127.5/-	129.3/-	127.9/-	131.51/-<br>130.83/-	129.3/-	126.8/-	128.3/-	
C_2/H_2	117.6/6.74	117.8/6.80	116.3/6.74	117.6/6.70	116.1/6.64	118.09/6.83<br>117.39/6.48	117.7/6.77	117.2/6.64	118.4/-	
C_3/H_3	146.2/-	146.4/-	144.7/-	146.2/-	143.9/-	145.40/-	146.1/-	145.8/-	147.5/-	

						144.99/-				
C_4/H_4	145.3/-	145.4/-	144.7/-	145.3/-	146.0/-	144.31/-<br>143.80/-	145.3/-	144.3/-	143.7/-	
C_5/H_5	116.3/6.69	116.9/6.66	114.6/6.60	116.3/6.66	114.9/6.65	116.98/6.76<br>117.09/6.57	116.4/6.69	117.1/6.92	115.1/7.23	
C_6/H_6	121.8/6.60	122.0/6.72	121.7/6.71	121.8/6.57	120.4/6.53	122.66/6.67<br>122.21/6.11	121.8/6.63	120.7/7.34	120.5/7.04	
C_7/H_7	37.9/3.09,3.00	38.3/3.14,3.04	36.5/3.60,3.06	37.9/3.05,2.96	36.5/3.00,2.96	38.50/2.95,3.08<br>37.95/2.67,2.88	38.0/3.00,3.07	36.4/7.64	37.7/7.75	
C_8/H_8	74.6/5.19	75.4/5.24	73.1/5.25	74.7/5.14	73.3/5.14	77.92/4.93<br>78.44/4.83	74.8/5.17	73.3/6.43	74.5/6.56	
C_9/H_9	173.4/-	169.0/-	167.1/-	173.5/-	172.1/-	178.09/-<br>177.77/-	173.5/-	173.1/-	173.6/-	
C_{10}/H_{10}			51.5/3.74						52.6/3.68	
$C_{1''}/H_{1''}$	134.7/-	123.6/-	120.4/-	125.3/7.28	121.9/-	133.67/-	133.9/-	131.1/-	133.1/-	133.5/-
$C_{2''}/H_{2''}$	114.1/6.87	113.6/-	112.0/-	117.5/6.82	142.3/-	116.18/6.58	113.5/6.80	113.1/6.65	114.8/6.74	113.6/6.68
$C_{3''}/H_{3''}$	146.5/-	146.1/-	144.0/-	125.0/-	108.3/6.84	145.30/-	146.6/-	144.9/-	145.3/-	144.7/-

$C_{4''}/H_{4''}$	146.5/-	145.2/-	143.7/-	109.7/6.80	150.8/-	144.08/-	146.7/-	144.4/-	143.4/-	144.7/-
$C_{5''}/H_{5''}$	116.3/6.76	111.9/-	110.5/-	147.3/-	146.9/-	116.90/6.57	116.4/6.76	114.8/6.63	116.3/6.82	116.2/7.14
$C_{6''}/H_{6''}$	118.9/6.78	118.9/-	117.6/-	148.3/-	114.1/6.63	120.17/6.20	118.4/6.72	117.6/6.51	116.0/6.81	117.8/6.82
$C_{7''}/H_{7''}$	86.2/5.71	159.6/-	158.5/-	115.5/6.59	131.4/6.87	40.38/4.45	88.8/5.92	87.5/5.81,4.33	88.4/6.34	88.0/5.85
$C_{8''}/H_{8''}$	39.3/3.74,3.26	99.5/-	97.1/-	152.3/-	123.6/6.84	47.41/4.28	57.6/4.38	56.0/-	57.1/4.47	57.2/4.31
$C_{9''}/H_{9''}$						174.14/-	175.2/-	170.1/-	171..8/-	174.0/-
$C_{10''}/H_{10''}$								52.2/3.61	53.2/3.63	
Determination condition	125MHz, MeOH-d_4+0.1% TFA / 500MHz, MeOH-d_4+0.1% TFA	125MHz, CD_3OD / 600MHz, CD_3OD	150MHz, CD_3OD / 600MHz, CD_3OD	CD_3OD	125MHz, CD_3OD / 500Hz, DMSO-d_6	75MHz, D_2O/CD_3OD / 300MHz, D_2O/CD_3OD	125.77MHz CD_3OD / 500.16MHz CD_3OD	150MHz, CD_3OD / 600MHz, CD_3OD	150MHz, CD_3OD / 600MHz, CD_3OD	$(CD_3)_2CO$-D_2O(10：1) / $(CD_3)_2CO$-D_2O(10：1)
Reference	[61]	[62]	[63]	[64]	[65]	[66]	[67]	[63]	[63]	[68]

Table S11. NMR Spectroscopic Data (δ) of Compounds 92~102

No.	92	93	94	95	96	97	98	99	100	101	102
Caffeoyl											
$C_{1'}/H_{1'}$	124.55/-	123.2/-	124.58/-	125.9/-	124.4/-	123.7/-	123.8/-	130.7/-	131.2/-	127.2/-	126.1/-
$C_{2'}/H_{2'}$	126.07/-	148.2/-	126.39/-	127.5/-	128.5/-	126.4/-	126.3/-	116.2/7.06	118.0/6.91	114.5/6.94	114.9/-
$C_{3'}/H_{3'}$	146.16/-	145.4/-	149.14/-	149.6/-	143.5/-	142.9/-	142.9/-	143.5/-	148.6/-	146.2/-	144.8/-
$C_{4'}/H_{4'}$	148.57/-	116.9/6.77	145.12/-	146.5/-	148.5/-	147.7/-	147.7/-	143.3/-	151.1/-	151.0/-	147.1/-
$C_{5'}/H_{5'}$	118.18/6.90	121.3/7.11	118.34/6.83	118.6/6.96	115.9/7.01	115.0/6.85	115.0/6.85	117.8/6.87	118.1/6.67	117.7/6.92	116.5/6.52
$C_{6'}/H_{6'}$	121.61/7.24	123.2/-	121.88/7.15	124.7/6.99	121.4/7.40	118.4/7.31	118.4/7.29	122.8/7.04	124.8/6.75	125.3/7.17	118.4/7.27
$C_{7'}/H_{7'}$	142.84/7.62	142.4/7.53	143.85/7.54	145.0/7.33	143.6/7.56	143.7/7.41	143.7/7.41	145.1/7.45	148.2/7.32	146.7/7.48	143.6/7.42
$C_{8'}/H_{8'}$	117.14/6.28	115.5/6.25	116.58/6.30	117.7/6.14	115.9/6.28	113.9/6.27	114.0/6.27	115.7/6.30	118.0/6.14	114.8/6.17	119.9/6.24
$C_{9'}/H_{9'}$	166.64/-	166.7/-	167.98/-	170.4/-	167.1/-	166.0/-	165.9/-	168.1/-	171.4/-	167.2/-	166.0/-
Dan Shen Su<br>C_1/H_1	128.69/-<br>129.11/-	126.9/-<br>127.7/-	129.05/-<br>128.66/-	133.4/-<br>130.8/-	128.6/-<br>128.4/-	127.1/-	127.2/-	128.0/-	133.0/-	129.0/-	127.3/-
C_2/H_2	116.11/6.67<br>116.79/6.71	116.4/6.48<br>116.4/6.71	117.53/6.78<br>117.53/6.54	119.6/6.99<br>119.2/6.53	116.9/-<br>118.0/-	116.5/6.62	116.5/6.62	117.0/6.76	119.9/6.91	117.2/6.72	117.0/-
C_3/H_3	145.95/-	143.8/-	145.17/-	146.6/-	145.2/-	143.9/-	143.9/-	145.1/-	146.5/-	146.1/-	142.9/-

C_4/H_4	144.63/-<br>145.52/-<br>144.36/-	144.8/-<br>143.7/-<br>144.6/-	145.26/-<br>146.47/-<br>145.92/-	146.3/-<br>145.6/-<br>145.2/-	144.8/-<br>144.1/-<br>144.9/-	144.8/-	144.9/-	148.1/-	145.3/-	145.2/-	143.9/-
C_5/H_5	116.08/6.65<br>117.31/6.74	114.9/6.56<br>114.9/6.64	116.45/6.69<br>116.36/5.59	119.1/6.92<br>118.8/6.66	116.0/6.60-<br>6.90<br>115.3/-	115.5/6.63	115.5/6.63	116.9/6.69	118.6/6.87	116.0/6.67	115.4/6.63
C_6/H_6	121.71/6.45<br>121.80/6.70	120.7/6.33<br>120.8/6.57	122.27/6.64<br>121.73/6.32	124.6/6.79<br>123.9/6.25	121.5/-<br>121.8/-	120.0/6.47	120.1/6.45	121.3/6.58	124.76.79	121.5/6.58	122.7/6.42
C_7/H_7	37.41/3.06<br>37.05/3.06	36.1/2.54,2.45<br>36.4/3.02,2.98	37.79/3.00<br>37.48/3.00	39.7/3.15,3.06<br>38.3/3.04,2.75	36.9/7.97<br>37.0/-	36.0/2.85,2.91	36.1/2.83,2.91	37.7/2.83,3.01	39.6/3.00,3.14	36.9/2.96,3.06	36.1/2.89,2.89
C_8/H_8	73.87/5.21<br>74.87/5.22	74.4/4.35<br>73.3/5.11	74.72/5.16<br>75.26/4.69	77.1/5.32<br>77.3/5.20	73.7/-<br>74.4/-	72.8/4.93	72.9/4.92	76.2/4.90	79.2/5.05	73.9/5.13	73.0/4.90
C_9/H_9	171.63/-<br>170.77/-	171.0/-<br>171.2/-	173.36/-<br>52.67/3.64	174.9/-<br>175.6/-	172.7/-<br>171.9/-	170.6/-	170.6/-	176.3/-	179.9/-	172.6/-	170.8/-
C_{10}/H_{10}				62.6/4.08							
				14.4/1.13							
$C_{1''}/H_{1''}$	133.28/-	127.8/-	133.54/-	134.6/-	125.9/-	126.0/-	126.0/-	129.5/-	134.8/-	125.5/-	126.5/-

$C_{2''}/H_{2''}$	113.21/6.82	113.6/6.90	113.35/6.76	115.9/6.92	118.1/-	117.3/6.44	117.3/6.43	114.8/6.84	117.9/7.07	117.8/7.30	113.9/-
$C_{3''}/H_{3''}$	145.47/-	144.4/-	146.72/-	146.8/-	144.4/-	144.8/-	144.8/-	145.3/-	146.5/-	146.4/-	144.8/-
$C_{4''}/H_{4''}$	144.66/-	143.9/-	146.15/-	145.3/-	147.9/-	147.2/-	147.2/-	145.8/-	146.4/-	148.8/-	147.8/-
$C_{5''}/H_{5''}$	115.95/6.80	114.7/6.68	116.39/6.77	120.1/6.97	117.1/-	115.3/6.55	115.3/6.55	116.9/6.61	118.9/6.89	116.1/6.75	115.3/-
$C_{6''}/H_{6''}$	117.14/6.83	118.4/6.68	118.35/6.66	120.4/6.77	124.5/-	122.9/6.43	122.9/6.43	119.2/6.69	122.7/6.92	124.7/7.11	123.7/6.42
$C_{7''}/H_{7''}$	87.54/5.87	87.0/5.90	88.12/5.86	89.4/5.94	144.3/-	141.1/7.69	141.1/7.69	77.2/5.27	76.4/5.09	129.1/7.34	140.6/7.64
$C_{8''}/H_{8''}$	57.06/4.47	53.2/4.77	59.75/4.35	58.6/4.33	122.2/-	123.4/-	123.3/-	78.5/4.63	86.7/4.70	139.2/-	124.4/7.64
$C_{9''}/H_{9''}$	171.20/-	170.0/-	172.32/-	174.7/-	167.5/-	168.4/-	168.4/-	173.4/-	178.6/-	166.0/-	168.7/-
Determination condition	DMSO-d_6 / DMSO-d_6	100MHz, methanol-d_4 / 400MHz, methanol-d_4	500MHz, DMSO-d_6 / 600MHz, CDC_{13}	125MHz, D_2O / 500MHz, D_2O	DMSO / DMSO	125MHz, DMSO / 500MHz, DMSO	125MHz, DMSO / 500MHz, DMSO	125MHz, CD_3OD / 400MHz, CD_3OD	75MHz, D_2O / 300MHz, D_2O	100MHz, methanol-d_4 / 400MHz, methanol-d_4	DMSO / DMSO
Reference	[59]	[60]	[70]	[71]	[72]	[73]	[73]	[74]	[75]	[76]	[72]

Table S12. NMR Spectroscopic Data (*δ*) of Compounds 103~113

No.	103	104	105	106	107	108	109	110	111	112	113
Caffeoyl C_1/H_1	129.3/-	130.87/-	127.8/-	126.30/-	126.34/-	126.40/-	127.9/-	127.9/-	122.7/-	127.7/-	127.2/-
C_2/H_2	116.5/7.12	115.17/7.13	115.0,115.1/7.04	115.62/7.13	115.50/7.00	115.67/6.98	115.5/7.05	115.3/7.07	114.7/6.93	115.2/7.05	116.1/7.08
C_3/H_3	147.6/-	147.84/-	146.7/-	122.79/-	146.39/-	146.42/-	149.5/-	149.6/-	151.6/-	146.8/-	146.0/-
C_4/H_4	147.2/-	148.01/-	149.7/-	115.75/-	149.24/-	149.26/-	146.8/-	146.8/-	151.2/-	149.6/-	148.8/-
C_5/H_5	115.2/7.10	117.49/7.22	116.6/6.78	123.21/7.07	116.66/6.65	116.67/6.72	116.5/6.78	116.6/6.79	117.9/6.72	116.2/6.77	114.5/6.80
C_6/H_6	121.0/7.06	121.41/7.06	123.0/6.94	116.49/6.87	122.40/6.90	122.25/6.94	123.0/6.95	123.1/6.97	120.3/6.72	123.0/6.94	122.6/6.95
C_7/H_7	144.0/7.43	134.25/7.58	147.1,147.2/7.56	148.15/7.68	114.71/6.24	146.22/7.43	169.1/-	168.5/-	142.5/7.98	147.2/7.57	114.9/7.59
C_8/H_8	118.0/6.31	114.61/6.34	115.2/6.27	113.45/6.34	146.12/7.42	114.87/6.22	115.2/6.33	115.3/6.29	117.7/6.55	115.0/6.29	146.8/6.30
C_9/H_9	168.3/-	168.59/-	169.3/-	167.84/-	166.44/-	166.79/-	147.0/7.59	147.1/7.57	169.2/-	169.0/-	168.7/-
Glc $C_{1'}/H_{1'}$	102.0/4.77	102.91/4.88	94.1,98.3/5.11,4.51	94.14/5.58	103.80/-	103.64/4.23	104.3/4.42	102.4/4.51	103.7/4.73	99.4/4.44	103.7/4.28
$C_{2'}/H_{2'}$	73.7/3.50	74.16/3.47-3.59	73.8,76.3/3.17-4.50	68.82/3.39-3.83	74.21/-	74.47/-	73.6/3.39	76.3/4.81	74.9/3.45	80.8/3.19	74.2/3.20

$C_{3'}/H_{3'}$	76.2/3.50	76.91/3.47-3.59	75.6,78.0/3.17-4.50	69.63/3.39-3.83	77.32/-	75.45/-	79.0/5.04	75.3/3.57	77.8/3.48	74.9/3.62	77.3/3.33-3.58
$C_{4'}/H_{4'}$	70.2/3.39	70.65/3.47-3.59	72.1,71.9/3.17-4.50	72.24/3.39-3.83	70.92/-	72.17/4.59	69.9/3.54	71.9/3.40	71.7/3.42	72.0/3.46	71.0/3.33-3.58
$C_{5'}/H_{5'}$	77.7/3.50	77.76/3.47-3.59	70.9,74.9/3.17-4.50	75.49/3.39-3.83	74.40/-	74.94/-	77.8/3.38	78.1/3.32	75.4/3.67	77.2/3.72	74.8/3.33-3.58
$C_{6'}/H_{6'}$	61.7/3.45,3.44	61.76/3.75-3.94	64.9,65.0/3.17-4.50	77.01/3.39-3.83	64.37/5.12,4.98	61.71/-	62.4/3.71,3.88	62.7/3.70,3.89	64.7/4.36,4.54	64.5/4.33,4.55	64.2/4.33,4.49
$C_{1''}/H_{1''}$					130.17/-	130.11/-	131.5/-	131.6/-	153.7/-	129.9/-	68.7/-
$C_{2''}/H_{2''}$					117.11/6.55	117.16/6.57	116.3/6.69	116.2/6.61	119.5/6.96	114.8/6.84	153.6/6.97
$C_{3''}/H_{3''}$					145.76/-	145.81/-	144.7/-	144.6/-	116.6/6.68	146.5/-	127.6/6.09
$C_{4''}/H_{4''}$					144.28/-	144.35/-	146.1/-	146.0/-	152.3/-	146.3/-	187.3/-
$C_{5''}/H_{5''}$					116.32/6.52	116.33/6.59	117.1/6.67	117.1/6.58	116.6/6.68	116.5/6.73	127.6/6.09
$C_{6''}/H_{6''}$					120.38/6.42	120.42/6.46	121.3/6.56	121.4/6.48	119.5/6.96	119.5/6.70	153.6/6.97
$C_{7''}/H_{7''}$					71.06/-	71.03/-	72.2/3.73,4.04	71.8/3.64,3.99		78.7/4.53	65.6/3.94,3.66
$C_{8''}/H_{8''}$					35.99/-	35.91/-	36.6/2.78	36.6/2.67		72.8/3.65,3.93	40.4/2.05
Determination	125MHz,	75.46MHz,	100MHz,	75MHz,	75MHz,	75MHz,	CD_3OD	CD_3OD	125MHz,	100.6MHz	100MHz,

condition	CD_3OD / 500MHz, CD_3OD	CD_3OD / 300.13MHz, CD_3OD	methanol-d_4 / 400MHz, CD_3OD	$CDCl_3$ / 300MHz, $CDCl_3$	DMSO-d_6 / 300MHz, DMSO-d_6	DMSO-d_6 / 300MHz, DMSO-d_6	/ CD_3OD	/ CD_3OD	CD_3OD / 500MHz, CD_3OD	CD_3OD / CD_3OD	CD_3OD / 400MHz, CD_3OD
Reference	[77]	[78]	[79]	[78]	[80]	[80]	[81]	[81]	[82]	[83]	[84]

Table S13. NMR Spectroscopic Data (δ) of Compounds 114~124

No.	114	115	116	117	118	119	120	121	122	123	124
Caffeoyl											
C_1/H_1	169.1/-	127.6/-	125.4/-	125.5/-	127.4/-	127.7/-	127.6/-	127.8/-	127.6/-	127.7,127.8/-	127.7/-
C_2/H_2	114.8/6.28	115.1/7.05	114.8/7.07	114.7/7.04	115.1/7.04	115.2/7.04	115.2/7.07	115.3/7.07	115.2/7.09	114.4,114.9/7.05,7.06	114.8/7.04
C_3/H_3	147.2/7.56	146.9/-	145.5/-	145.4/-	147.0/-	146.8/-	146.8/-	147.0/-	146.9/-	146.8,147.3/-	149.6/-
C_4/H_4	127.7/-	149.7/-	148.3/-	148.0/-	150.1/-	149.7/-	149.8/-	149.9/-	149.8/-	149.6,149.9/-	147.3/-
C_5/H_5	115.2/7.03	116.6/6.78	115.7/6.76	115.6/6.77	116.6/6.76	117.1/6.77	116.5/6.80	116.6/6.79	116.6/6.81	116.6/6.77,6.78	116.4/6.72
C_6/H_6	146.8/-	123.1/6.96	121.3/6.97	121.3/7.01	123.1/6.94	123.0/6.95	123.1/6.97	123.3/7.97	123.2/6.99	123.1,123.3/6.95,6.96	123.2/6.91
C_7/H_7	149.6/-	147.2/7.57	145.2/7.47	113.8/6.23	147.3/7.56	147.3/7.57	147.6/7.58	147.7/7.60	147.7/7.62	147.3,148.5/7.57,7.66	146.4/7.57
C_8/H_8	116.5/6.76	114.9/6.29	113.2/6.29	145.5/7.49	114.8/6.27	115.1/6.28	114.5/6.32	114.6/6.32	114.5/6.34	115.3,115.4/6.29,6.32	115.4/6.29
C_9/H_9	123.1/6.93	169.0/-	165.4/-	165.6/-	168.8/-	168.5/-	168.9/-	169.1/-	169.0/-	167.2,169.2/-	169/-
Glc											
$C_{1'}/H_{1'}$	99.2/4.59	99.4/4.70	102.4/4.31	102.2/4.37	104.3/4.31	104.5/4.33	98.9/4.70	99.8/4.80	99.7/4.82	95.8/5.60	99.9/4.73
$C_{2'}/H_{2'}$	74.4/3.25	74.8/3.25-3.27	73.6/3.18	73.8/3.21	75.0/3.22	75.0/3.18	73.8/3.65-3.67	75.0/3.28	74.9/3.30	74.1/3.43-3.50	74.8/3.30
$C_{3'}/H_{3'}$	77.4/3.39	77.6/3.38-3.40	81.3/3.41	78.8/3.69	77.9/3.36	78.1/3.33	78.1/3.29-3.31	77.8/3.42	77.7/3.44	78.0/3.43-3.50	77.8/3.40
$C_{4'}/H_{4'}$	71.7/3.40	71.7/3.38-3.40	68.4/3.24	69.3/4.64	71.5/3.30	71.5/3.30	71.6/3.28-3.30	71.9/3.28	71.8/3.29	71.4/3.43-3.50	71.7/3.39

$C_{5'}/H_{5'}$	75.7/3.54-3.57	75.8/3.50-3.52	73.4/3.49	72.7/3.65	78.0/3.29	78.1/3.28	74.7/3.15-3,17	78.8/3.30	78.6/3.37	76.4/3.68	75.7/3.55
$C_{6'}/H_{6'}$	64.5/4.48,4.35	64.1/4.46,3.40	63.2/4.20,4.39	67.0/3.42	62.7/3.65,3.85	62.7/3.65,3.82	62.7/3.88,3.67	63.1/3.93,3.65	63.0/3.67,3.95	64.4/4.50,4.32	64/4.40,4.53
$C_{1''}/H_{1''}$	93.1/5.65	97.9/4.60	129.1/-	129.1/-	114.6/5.25,5.34	113.3/5.25,5.23	93.1/5.67	95.2/5.16	95.1/5.19		96/5.33
$C_{2''}/H_{2''}$	142.5/6.32	63.0/3.83-3.85,3.39-3.41	116.2/6.61	115.3/6.64	145.4/-	147.1/-	95.9/5.32	142.5/6.37	142.4/6.39		152.4/7.41
$C_{3''}/H_{3''}$	108.4/4.93	23.9/1.73-1.75,1.56	144.9/-	144.6/-	72.3/4.42	74.6/5.58	34.4/2.44-2.46,2.36-2.38	103.1/4.98	103.0/5.00		115.9/-
$C_{4''}/H_{4''}$	72.8/-	38.3/2.00	143.5/-	143.2/-	74.0/3.91,3.76	71.9/4.12,3.85	34.7/2.09	36.9/2.60	36.8/2.60		32.6/3.06
$C_{5''}/H_{5''}$	78.3/3.67	73.3/4.01	115.4/6.58	116.2/6.63	65.4/4.79,4.73	63.9/4.18,4.14	87.7/5.01	81.4/5.03	81.3/5.04		39.5/1.92,2.17
$C_{6''}/H_{6''}$	46.9/1.85,1.75	62.0/3.39-3.41	119.4/6.46	119.5/5.49			70.4/4.44	60.4/3.70	60.3/3.72		82.3/4.90
$C_{7''}/H_{7''}$	78.4/-	66.0/-	35.1/2.69,2.71	70.2/3.81			79.9/-	67.0/-	66.9/-		43.1/2.42
$C_{8''}/H_{8''}$	59.5/2.54	43.3/2.28	70.4/3.61,3.79	34.9/2.70			48.3/2.62	43.3/2.62	43.2/2.65		43.4/2.48
$C_{9''}/H_{9''}$	25.0/1.18	61.5/4.07,3.64					62.1/4.04,3.68	61.4/4.17,3.84	61.3/3.85,4.19		14.4/1.08
$C_{1'''}/H_{1'''}$			109.2/5.23	109.4/5.24 109.0/4.79							170.6/-
$C_{2'''}/H_{2'''}$			76.0/3.82	75.8/3.70							

C$_{3'''}$/H$_{3'''}$			79.0/-	75.7/3.70<br>78.6/-<br>78.1/-							
C$_{4'''}$/H$_{4'''}$			73.6/3.63,4.01	73.6,<br>73.3/3.60-<br>3.80							
C$_{5'''}$/H$_{5'''}$			63.6/3.40	63.6,<br>62.9/3.18-<br>3.30							
Determination condition	100MHz, CD$_3$OD / 400MHz, CD$_3$OD	150MHz, CD$_3$OD / 600MHz, CD$_3$OD	100MHz, DMSO-d$_6$ / 400MHz, DMSO-d$_6$	100MHz, DMSO-d$_6$ / 400MHz, DMSO-d$_6$	125MHz, CD$_3$OD / 500MHz, CD$_3$OD	125MHz, CD$_3$OD / 500MHz, CD$_3$OD	150MHz, CD$_3$OD / 600MHz, CD$_3$OD	150MHz, CD$_3$OD / 600MHz, CD$_3$OD	CD$_3$OD / CD$_3$OD	100MHz, methanol-d$_4$ / 400MHz, CD$_3$OD	75MHz, CD$_3$OD / 300MHz, CD$_3$OD
Reference	[85]	[86]	[87]	[88]	[89]	[89]	[86]	[90]	[91]	[79]	[92]

Table S14. NMR Spectroscopic Data (*δ*) of Compounds 125~135

No.	125	126	127	128	129	130	131	132	133	134	135
Caffeoyl											
C_1/H_1	127.7/-	127.0/-	127.6/-	125.3/-<br>128.6/-	128.9/-<br>128.5/-	128.4/-<br>128.6/-	125.2/-	127.65,127.69/-	126.9/-	127.6/-	127.8/-
C_2/H_2	115.1/7.04	115.8/-	115.1/-	114.9/7.06<br>115.1/7.18	115.1/7.12<br>114.9/7.19	115.1/7.18<br>115.1/7.18	113.3/7.06	115.21/7.07,7.06	115.7/7.60	114.7/7.07	114.8/6.6-7.1
C_3/H_3	149.6/-	147.1/-	146.9/-	145.6/-<br>146.8/-	146.9/-<br>146.8/-	146.8/-<br>146.8/-	145.4/-	146.82/-	146.8/-	149.7/-	149.8/-
C_4/H_4	146.7/-	150.4/-	149.7/-	148.7/-<br>147.5/-	146.9/-<br>147.5/-	147.7/-<br>147.5/-	148.5/-	149.74,149.78/-	147.5/-	146.7/-	146.9/-
C_5/H_5	116.5/6.77	116.5/-	116.2/-	115.8/6.77<br>116.1/7.12	115.9/7.08<br>116.0/7.12	116.1/7.12<br>116.1/7.12	115.7/6.75	116.51/6.79	116.6/7.14	116.5/6.79	116.6/6.6-7.1
C_6/H_6	123.2/6.88	122.2/-	123.2/-	121.7/7.02<br>120.9/7.12	120.3/6.96<br>120.8/7.12	121.2/7.12<br>120.9/7.12	121.6/7.02	123.24/6.97,6.96	122.2/7.17	123.2/6.95	123.2/6.6-7.1
C_7/H_7	169.1/-	147.6/8.04	147.3/7.61	146.6/7.57<br>144.8/7.52	143.5/7.38<br>144.7/7.53	146.0/7.62<br>144.8/7.52	146.3/7.55	147.90,148.01/7.60	114.7,114.6/6.69	168.3/-	168.3/-
C_8/H_8	114.9/6.28	115.2/6.83	114.7/6.35	113.2/6.26	117.5/6.24	115.3/6.45	114.8/6.25	114.70,114.85/6.29,6.28	146.4/8.00	115.3/6.28	115.4/-

				115.8/6.45	115.8/6.45	115.8/6.44					
C_9/H_9	147.2/7.55	167.1/-	168.5/-	165.2/-<br>166.3/-	167.7/-<br>166.2/-	165.1/-<br>166.3/-	165.0/-	168.43,168.33/-	167.0/-	148.0/7.60	148.0/7.59
Glc<br>$C_{1'}/H_{1'}$	104.0/4.38<br>105.3/4.58	104.0/4.84<br>106.7/4.88	104.5/4.39<br>105.8/4.46	94.1/5.50<br>101.6/4.78	101.2/4.86<br>101.5/4.80	94.2/5.52<br>101.4/4.78<br>101.5/4.80	94.1/5.42	94.04,98.16/5.13,4.56	93.8,98.4/5.71,5.21	104.2/4.39	104.3/4.37
$C_{2'}/H_{2'}$	74.4/3.43<br>75.5/3.29	76.3/-<br>75.9/-	75.5/-<br>76.1/6.60-7.20	72.4/3.20-3.38<br>75.8/3.20-3.38	75.5/3.30<br>75.7/3.32	72.4/3.17-3.39<br>75.8/3.17-3.39<br>75.8/3.17-3.39	72.5/-	76.12,77.38/3.56,3.38	75.0,77.4/4.20-4.25,4.06-4.11	76.1/-	76.3/-
$C_{3'}/H_{3'}$	87.7/3.58<br>77.8/3.41	84.8/-<br>78.1/-	84.1/-<br>77.9/6.60-7.20	76.1/3.20-3.38<br>73.2/3.20-3.38	73.9/3.71<br>73.3/3.32	76.1/3.17-3.39<br>73.2/3.17-3.39<br>73.2/3.17-3.39	76.3/-	79.21.81.73/4.06,3.81	78.0,80.4/4.87-4.89,4.48-4.51	81.6/-	81.7/-
$C_{4'}/H_{4'}$	70.3/3.47<br>71.6/3.29	71.5/-<br>71.3/-	70.9/-<br>71.6/6.60-7.20	69.6/3.20-3.38<br>69.8/3.20-3.38	69.7/3.20<br>69.9/3.30	69.6/3.17-3.39<br>69.8/3.17-3.39<br>69.8/3.17-3.39	70.6/-	70.70,70.80/4.94,4.93	70.7,70.9/5.59-5.62,4.90-4.92	70.4/4.92	70.4/-
$C_{5'}/H_{5'}$	75.0/3.58<br>78.1/3.35	76.2/-<br>78.3/-	75.2/	74.6/3.58<br>77.2/3.20-3.38	73.2/3.30<br>77.1/3.32	74.6/3.51<br>77.2/3.17-3.39	76.3/-	71.23,74.71/4.02,3.58	69.9,74.4/4.93-4.96,4.15-4.18	76.0/-	76.1/-

			78.1/6.60-7.20			77.2/3.17-3.39					
$C_{6'}/H_{6'}$	64.6/4.34,4.52<br>62.6/3.65,3.90	62.7/-<br>62.1/-	62.4/<br>62.7/6.60-7.20	63.4/4.41,4.16<br>60.7/3.71,3.47	63.3/4.23,4.46<br>60.7/3.48,3.71	63.4/4.41,4.16<br>60.7/3.70,3.47<br>60.7/3.73,3.47	66.5/-	62.37,62.50/3.18,3.49	68.6/3.94-3.99,4.28-4.34	62.4/-	62.5/-
$C_{1''}/H_{1''}$	131.4/-	130.3/-	133.8/-						111.0/5.64	139.9/-	130.8/-
$C_{2''}/H_{2''}$	117.1/6.68	116.7/-	114.8/-						77.7,77.8/4.74	130.0/7.25	116.2/6.6-7.1
$C_{3''}/H_{3''}$	144.6/-	146.3/-	146.2/-						80.3/-	129.3/7.25	130.9/6.6-7.1
$C_{4''}/H_{4''}$	146.1/-	145.6/-	146.0/-						75.0/4.47-4.52,4.23-4.27	127.2/7.25	156.8/-
$C_{5''}/H_{5''}$	116.4/6.64	117.5/-	116.6/-						65.4,65.3/4.08-4.10	129.3/7.25	130.8/6.6-7.1
$C_{6''}/H_{6''}$	121.3/6.54	120.5/-	119.1/-							130.0/7.25	116.2/6.6-7.1
$C_{7''}/H_{7''}$	72.4/3.72,3.96	36.1/2.96	73.5/-							71.8/-	72.4/-

$C_{8''}/H_{8''}$	36.7/2.78	74.6/-	76.1/-							37.2/2.94	36.4/2.84
Rhamnose											
$C_{1'''}/H_{1'''}$							100.5/4.46	103.03,103.12/5.20,5.15	102.8,102.9/6.30,6.27	103.0/5.20	103.0/5.19
$C_{2'''}/H_{2'''}$							70.3/-	72.32/3.94	72.5,72.6/4.84-4.86,4.78-4.80	72.1/-	72.3/-
$C_{3'''}/H_{3'''}$							70.0/-	72.06/3.61	72.4/4.39-4.45	72.3/-	72.2/-
$C_{4'''}/H_{4'''}$							71.9/-	73.78,73.82/3.32,3.31	73.9/4.19-4.23	73.8/-	73.9/-
$C_{5'''}/H_{5'''}$							68.4/-	70.38,70.70/3.58,3.57	70.1,69.8/4.44-4.52	70.6/-	70.7/-
$C_{6'''}/H_{6'''}$							18.0/-	18.48/1.10	18.9,19.0/1.62,1.53	18.5/1.10	18.4/1.09
Determination condition	CD_3OD / CD_3OD	125MHz pyridine-d_5 / 600MHz pyridine-d_5	22.5MHz CD_3OD / $CDCl_3$	100MHz, DMSO-d_6 / 400MHz, DMSO-d_6	100MHz, DMSO-d_6 / 400MHz, DMSO-d_6	100MHz, DMSO-d_6 / 400MHz, DMSO-d_6	DMSO-d_6 / DMSO-d_6	100MHz CD_3OD / 400MHz CD_3OD	100MHz, C_5D_5N / 400MHz, C_5D_5N	50.10MHz, CD_3OD / 200.40MHz, CD_3OD	125MHz, CD_3OD / 500MHz, CD_3OD
Reference	[81]	[93]	[94]	[95]	[95]	[95]	[96]	[97]	[98]	[99]	[100]

Table S15. NMR Spectroscopic Data (*δ*) of Compounds 136~146

No.	136	137	138	139	140	141	142	143	144	145	146
Caffeoyl											
C_1/H_1	125.4/-	125.5/-	128.37/-	125.6/-	127.68/-	127.68/-	125.5/-	127.66/-	127.7/-	127.7/-	127.7/-
C_2/H_2	114.8/7.05	114.8/7.06	115.96/7.04	114.9/7.04	115.10/7.03	115.26/7.06	115.0/7.03	115.24/7.05	114.8/6.6-7.2	114.8/7.06	114.7/6.5-7.2
C_3/H_3	145.6/-	145.6/-	147.36/-	145.5/-	146.73/-	146.85/-	145.5/-	146.79/-	149.6/-	149.7/-	149.6/-
C_4/H_4	148.6/-	148.5/-	150.10/-	148.2/-	149.57/-	149.82/-	145.0/-	149.74/-	146.7/-	146.8/-	146.6/-
C_5/H_5	115.8/6.76	115.8/6.76	117.04/6.76	115.7/6.75	116.54/6.76	116.56/6.78	115.8/6.75	116.5/6.77	116.2/6.6-7.2	116.6/6.78	116.4/6.5-7.2
C_6/H_6	121.4/7.01	121.3/7.01	123.39/6.94	121.3/7.01	123.13/6.88	123.27/6.95	121.4/6.90	123.19/6.95	123.2/6.6-7.2	123.2/6.95	123.2/6.5-7.2
C_7/H_7	145.6/7.50	145.2/7.49	147.48/7.58	144.9/7.47	147.23/7.55	148.06/7.59	148.4/7.55	147.98/7.59	168.3/-	168.3/-	168.1/-
C_8/H_8	113.6/6.25	114.2/6.27	115.67/6.31	114.7/6.26	114.83/6.28	114.72/6.28	113.8/6.23	114.71/6.27	115.5/6.27	115.3/6.28	115.5/6.25
C_9/H_9	165.9/-	165.7/-	169.57/-	166.1/-	169.13/-	168.34/-	166.5/-	168.27/-	147.9/7.61	148.0/7.60	148.1/7.59
Glc											
$C_{1'}/H_{1'}$	102.9/4.31	100.2/4.49	104.89/4.40	102.7/4.34	104.35/4.32	104.22/4.42	101.3/4.32	104.18/4.37	104.1/4.40	104.2/4.38	101.6/4.50
$C_{2'}/H_{2'}$	73.0/3.41	73.4/4.64	74.03/3.36	71.4/3.18	75.65/3.32	76.22/3.46	77.6/3.37	76.18/3.39	76.0/-	76.0/-	75.1/-
$C_{3'}/H_{3'}$	73.5/3.10	74.1/3.42	79.49/5.02	77.5/4.88	83.96/3.52	81.70/3.84	82.1/3.78	81.62/3.81	81.6/-	81.6/-	80.3/-
$C_{4'}/H_{4'}$	71.0/4.66	70.7/3.44	70.56/3.51	68.1/3.28	70.37/3.41	70.60/3.43	70.5/3.56	70.58/4.91	70.3/-	70.4/4.92	70.7/-

$C_{5'}/H_{5'}$	73.9/3.45	75.5/3.38	77.23/3.51	75.1/3.42	75.38/3.55	75.92/3.56	73.5/3.66	76.00/3.54	76.1/-	76.2/-	76.0/-
$C_{6'}/H_{6'}$	66.1/3.53,3.33	66.6/3.84,3.48	68.35/3.63,3.97	66.5/3.82,3.49	64.61/4.35,4.49	64.66/4.33,4.54	63.3/4.53,4.62	62.35/3.52,3.62	62.4/-	62.4/-	62.2/-
Rhamnose											
$C_{1''}/H_{1''}$	100.5/4.50	100.7/4.60	102.75/4.74	100.7/4.59	102.68/5.17	103.07/5.26	102.4/5.22	102.99/5.18	102.8/5.20	103.0/5.20	103.1/5.16
$C_{2''}/H_{2''}$	70.6/3.59	70.5/3.63	72.89/3.84	70.6/3.62	72.38/3.95	72.37/3.92	72.3/3.90	72.33/3.91	72.1/-	72.1/-	72.0/-
$C_{3''}/H_{3''}$	70.6/3.36	70.3/3.45	72.72/3.72	70.4/3.62	72.24/3.69	70.29/3.59	73.3/4.9	72.05/3.58	72.0/-	72.3/-	71.7/-
$C_{4''}/H_{4''}$	71.9/3.57	72.0/3.19	74.56/3.36	71.9/3.44	73.98/3.39	75.53/4.78	75.4/4.7	73.78/3.29	73.8/-	73.8/-	73.6/-
$C_{5''}/H_{5''}$	68.4/3.34	68.4/3.46	70.36/3.67	68.4/3.48	70.02/3.97	67.17/3.54	67.7/3.60	70.38/3.56	70.7/-	70.6/-	70.7/-
$C_{6''}/H_{6''}$	17.8/1.05	18.0/1.14	18.54/1.26	17.9/1.13	17.85/1.24	18.4/1.10	17.8/1.06	18.42/1.09	18.4/1.11	18.5/1.10	18.4/1.07
$C_{7''}/H_{7''}$						20.5/1.96	20.6/1.92		56.6/3.86	56.6/3.81	20.9)/1.99
$C_{8''}/H_{8''}$						172.3/-	170.1/1.91				171.5/-
$C_{9''}/H_{9''}$							20.8/-				
$C_{10''}/H_{10''}$							171.9/-				
$C_{1'''}/H_{1'''}$	129.2/-	129.1/-	131.96/-	129.5/-	131.40/-	131.5/-	129.1/-	131.47/-	131.6/-	132.9/-	131.9/-
$C_{2'''}/H_{2'''}$	115.5/6.62	115.4/6.54	116.88/6.68	115.5/6.61	117.07/6.67	117.1/6.70	115.5/6.66	117.10/6.69	113.9/6.6-7.2	113.0/6.74	116.6/6.5-7.2
$C_{3'''}/H_{3'''}$	144.9/-	144.9/-	145.21/-	144.7/-	146.08/-	146.1/-	145.3/-	146.09/-	148.8/-	147.5/-	144.4/-
$C_{4'''}/H_{4'''}$	143.5/-	143.5/-	146.63/-	143.5/-	144.61/-	144.6/-	146.4/-	144.64/-	145.9/-	147.3/-	145.9/-

$C_{5'''}/H_{5'''}$	116.3/6.63	116.2/6.55	117.62/6.66	116.3/6.63	116.36/6.63	116.3/6.68	116.2/6.66	116.30/6.67	116.6/6.6-7.2	117.1/6.81	117.2/6.5-7.2
$C_{6'''}/H_{6'''}$	119.5/6.49	119.6/6.41	121.80/6.55	119.5/6.48	121.26/6.53	121.3/6.56	119.5/6.51	121.25/6.56	122.4/6.6-7.2	121.2/6.68	121.4/6.5-7.2
$C_{7'''}/H_{7'''}$	35.1/2.67	35.1/2.56	37.17/2.78	35.1/2.69	36.65/2.77	36.5/2.79	35.1/2.73	36.54/2.78	72.3/-	72.1/-	72.5/
$C_{8'''}/H_{8'''}$	70.3/3.83,3.61	69.8/3.76,3.54	72.83/3.68,3.97	70.2/3.82,3.62	72.32/3.72,4.00	72.2/3.72,4.04	70.3/3.66	72.22/3.71,4.03	36.6/2.88	36.5/2.82	36.2/2.69
Determination condition	100MHz, DMSO-d_6 / 400MHz, DMSO-d_6	100MHz, DMSO-d_6 / 400MHz, DMSO-d_6	125MHz, CD_3OD / 500MHz, CD_3OD	100MHz, DMSO-d_6 / 400MHz, DMSO-d_6	150MHz, CD_3OD / 600MHz, CD_3OD	125MHz, DMSO-d_6 / 300MHz, DMSO-d_6	125MHz, DMSO-d_6 / 300MHz, DMSO-d_6	150MHz, CD_3OD / 600MHz, CD_3OD	22.5MHz, Methanol-d_4 / 89.55MHz, Methanol-d_4	50.10MHz, CD_3OD / 200.40MHz, CD_3OD	22.5MHz, Methanol-d_4 / 89.55MHz, Methanol-d_4
Reference	[101]	[101]	[102]	[101]	[103]	[104]	[104]	[103]	[105]	[99]	[105]

Table S16. NMR Spectroscopic Data (*δ*) of Compounds 147~154

No.	147	148	149	150	151	152	153	154
Caffeoyl C_1/H_1	127.6/-	127.66/-	127.6/-	127.7/-	127.5/-	127.8/-	127.64/-	127.8/-
C_2/H_2	115.2/7.05	115.24/7.06	115.2/7.00	115.2/7.10	115.2/7.05	115.5/7.01	115.27/7.06	115.4/7.00
C_3/H_3	147.2/-	146.85/-	146.9/-	146.9/-	146.9/-	146.9/-	146.83/-	146.9/-
C_4/H_4	149.8/-	149.82/-	149.8/-	149.8/-	149.9/-	149.8/-	149.83/-	149.8/-
C_5/H_5	116.5/6.79	116.54/6.80	116.5/6.73	116.2/6.80	116.5/6.82	116.5/6.73	116.49/6.78	116.6/6.73
C_6/H_6	123.2/6.96	123.28/6.96	123.1/6.90	123.1/7.00	123.2/6.95	123.1/6.91	123.28/6.96	123.1/6.91
C_7/H_7	114.6/6.27	148.08/7.60	147.8/7.54	147.6/7.64	148.1/7.59	147.8/6.25	148.26/7.61	147.8/7.56
C_8/H_8	148.0/7.60	114.79/6.28	114.6/6.24	114.8/6.33	114.6/6.27	114.6/7.55	114.46/6.28	114.7/6.25
C_9/H_9	168.2/-	168.30,168.29/-	168.3/-	168.3/-	168.2/	168.2/-	167.97/-	168.1/-
Glc $C_{1'}/H_{1'}$	104.6/4.41	104.60,104.06/4.43,4.41	104.4/4.36	105.0/4.49	104.1/4.47	99.9/4.51	99.04/4.55	99.5/4.44
$C_{2'}/H_{2'}$	76.1/3.46	76.12,76.43/3.42	75.2/-	75.3/3.36	76.3/3.50-3.65	74.3/3.40	81.93/3.45	80.9/3.23
$C_{3'}/H_{3'}$	81.4/3.84	81.31,81.43/3.82	75.6/-	75.8/3.68	81.2/3.86	76.0/5.25	77.42/4.13	73.1/3.82
$C_{4'}/H_{4'}$	70.4/4.95	70.45/4.92	73.9/-	72.8/4.97	70.4/4.93	70.2/3.62	70.40/5.10	72.4/4.99

$C_{5'}/H_{5'}$	76.1/3.55	76.07,76.09/3.54	74.8/-	75/3.70	76.4/3.50-3.65	78.5/3.67	77.81/3.57,3.75	76.5/3.81
$C_{6'}/H_{6'}$	62.3/3.63,3.53	62.32/3.52,3.61	67.7/-	67.6/3.79,3.52	62.5/3.50-3.65	67.8/3.45,3.72	62.09/3.67	67.6/3.45,3.72
Rhamnose $C_{1''}/H_{1''}$	102.9/5.20	102.94,103.00/5.22,5.20	102.3/4.58	102.2/4.67	103.0/5.22	102.3/4.73	102.19/5.17	102.4/4.57
$C_{2''}/H_{2''}$	72.4/3.93	72.37/3.96	72.2/-	72.0/3.88	72.1/3.93	72.0/3.85	72.08/3.77	72/3.80
$C_{3''}/H_{3''}$	72.0/3.58	72.06/3.60	72.4/-	72.3/3.78	72.1/3.50-3.65	72.4/3.70	71.94/3.52	72.5/3.60
$C_{4''}/H_{4''}$	73.8/3.28	73.79/3.31	73.5/-	73.9/3.41	73.8/3.27-3.34	74.0/3.37	73.57/3.27	74/3.28
$C_{5''}/H_{5''}$	70.4/3.57	70.38/3.56	69.9/-	69.9/3.65	70.4/3.50-3.65	69.9/3.71	70.16/-	69.9/3.51
$C_{6''}/H_{6''}$	18.5/1.10	18.48/1.07	18.0/1.14	18.0/1.24	18.4/1.09	18.0/1.26	18.32/1.11	17.9/1.14
$C_{1'''}/H_{1'''}$	133.6/-	133.60,133.87/-	133.8/-	131.8/-	127.6/-	129.8/-	129.83/-	129.9/-
$C_{2'''}/H_{2'''}$	115.7/6.76	114.65,114.67/6.85	114.7/6.80	115.0/6.79	115.5/7.42	115.1/6.78	2 114.50/6.83	115.1/6.79
$C_{3'''}/H_{3'''}$	146.2/-	146.12,146.03/-	146.1/-	146.5/-	152.7/-	146.3/-	146.37/-	146.3/-
$C_{4'''}/H_{4'''}$	146.0/-	146.31,146.26/-	146.2/-	146.3/-	146.9/-	146.6/-	146.41/-	146.6/-
$C_{5'''}/H_{5'''}$	116.1/6.79	116.21,116.17/6.77	116.2/6.66	116.5/6.82	116.1/6.77	116.4/6.69	116.23/6.73	116.2/6.67
$C_{6'''}/H_{6'''}$	118.9/6.74	119.00,119.11/6.56	119.1/6.62	119.7/6.70	123.0/7.43	119.5/6.66	118.90/6.69	119.4/6.67
$C_{7'''}/H_{7'''}$	76.7/3.98	73.57,74.21/4.75	76.5/4.70	81.9/4.48	196.2/-	78.7/4.50	78.43/4.60	78.8/4.51
$C_{8'''}/H_{8'''}$	74.2/4.75	76.18,76.69/3.95,3.60;3.83,3.71	73.4/-	75.4/4.02,3.66	72.1/4.92,5.26	72.6/3.90,3.61	72.96/3.60,3.99	72.8/3.91,3.62

$C_{9'''}/H_{9'''}$				69.6/3.42				
$C_{10'''}/H_{10'''}$				32.9/1.60				
$C_{11'''}/H_{11'''}$				20.4/1.40				
$C_{12'''}/H_{12'''}$				14.7/0.94				
Determination condition	125MHz, CD_3OD / 500MHz, CD_3OD	100MHz, CD_3OD / 400MHz, CD_3OD	125MHz, CD_3OD / 500MHz, CD_3OD	125MHz, CD_3OD / 500MHz, CD_3OD	125MHz, CD_3OD / 500MHz, CD_3OD	100MHz, CD_3OD / 400MHz, CD_3OD	125MHz, CD_3OD / 500MHz, CD_3OD	125MHz, CD_3OD / 500MHz, CD_3OD
Reference	[106]	[97]	[107]	[107]	[108]	[109]	[110]	[107]

Table S17. NMR Spectroscopic Data (*δ*) of Compounds 155~162

No.	155	156	157	158	159	160	161	162
Caffeoyl C_1/H_1	127.6/-	127.6/-	127.1/-	127.6/-	128.0/-	127.6/-	127.6/-	127.39/-
C_2/H_2	115.2/7.08	115.2/7.06	115.7/6.63-7.05	114.2/7.07	115.6/7.06	115.4/7.05	115.2/7.06	115.35/7.06
C_3/H_3	146.9/-	146.8/-	144.9/-	147.0/-	147.1/-	146.8/-	146.8/-	146.42/-
C_4/H_4	149.4/-	149.7/-	147.9/-	150.2/-	150.0/-	149.8/-	149.8/-	149.55/-
C_5/H_5	116.3/6.81	116.3/6.79	114.2/6.63-7.05	114.6/6.80	116.9/6.78	116.6/6.78	116.5/6.78	116.69/6.78
C_6/H_6	123.4/6.98	123.2/6.99	123.4/6.63-7.05	123.4/6.97	123.5/6.96	123.2/6.96	123.2/6.96	123.51/6.96
C_7/H_7	114.6/6.26	168.3/-	117.2/6.29	117.1/6.25	148.3/7.59	148.1/7.60	114.7/6.28	114.35/6.27
C_8/H_8	148.0/7.59	114.7/6.28	147.9/7.60	148.0/7.59	115.1/6.28	114.7/6.27	148.0/7.60	148.30/7.59
C_9/H_9	168.2/-	148.0/7.59	168.4/-	127.6/-	168.4/-	167.9/-	168.1/-	168.54/-
Glc $C_{1'}/H_{1'}$	104.1/4.38	104.1/4.37	102.7/4.28	104.2/4.38	104.5/4.37	101.7/4.53	104.1/4.37	103.74/4.36
$C_{2'}/H_{2'}$	76.4/3.41	76.0/3.39	74.3/3.29-3.96	80.0/3.66	76.5/3.38	75.1/4.86	76.1/3.39	75.53/3.34
$C_{3'}/H_{3'}$	80.5/3.83	81.9/3.78	81.1/3.29-3.96	80.4/3.85	81.9/3.80	80.4/4.00	81.7/3.80	82.22/3.74
$C_{4'}/H_{4'}$	70.4/4.93	70.6/4.92	70.8/3.29-3.96	70.5/4.93	71.2/4.94	71.0/5.02	70.8/4.93	70.37/4.93

$C_{5'}/H_{5'}$	75.9/3.55	75.9/3.53	72.7/3.29-3.96	76.4/3.51-3.62	74.9/3.69	74.7/3.74	74.6/3.71	73.90/3.71
$C_{6'}/H_{6'}$	62.3/3.65,3.55	62.3/3.54,3.63	67.3/3.29-3.96	62.4/3.51-3.62	68.8/3.74,3.49	68.2/3.74,3.50	68.4/3.72,3.48	68.11/3.47,3.70
$C_{7'}/H_{7'}$						171.5/-		
$C_{8'}/H_{8'}$						20.8/1.98		
Rhamnose								
$C_{1''}/H_{1''}$	102.1/5.28	101.9/5.36	102.0/5.05	102.1/5.28	103.3/5.18	103.2/4.79	102.9/5.17	101.92/5.34
$C_{2''}/H_{2''}$	72.4/3.90-3.85	79.9/3.90	70.4/3.29-3.96	72.6/3.60,3.65	72.6/3.91	72.6/3.62	72.3/4.07	79.76/3.88
$C_{3''}/H_{3''}$	72.3/3.67	71.6/3.66	69.8/3.29-3.96	72.5/3.29.3.90	72.4/3.58	71.9/3.50	71.9/3.73	71.17/3.62
$C_{4''}/H_{4''}$	80.0/3.41	74.1/3.25	70.8/3.29-3.96	75.9/3.30	74.1/3.29	73.6/3.26	80.3/3.58	73.67/3.25
$C_{5''}/H_{5''}$	68.8/3.55	70.3/3.54	69.7/3.29-3.96	76.4/3.60-3.95	70.7/3.57	70.7/3.50	70.4/3.54	70.37/3.43
$C_{6''}/H_{6''}$	18.7/1.12	18.5/1.08	17.6/1.01	18.7/1.12	18.7/1.09	18.4/1.07	18.5/1.09	18.27/1.07
$C_{1'''}/H_{1'''}$	131.4/-	131.5/-	131.8/-	131.5/6.69	131.8/-	131.8/-	131.4/-	131.45/-
$C_{2'''}/H_{2'''}$	116.6/6.69	117.1/6.70	116.8/6.63-7.05	115.2/-	117.4/6.70	117.2/6.64	117.1/6.69	117.25/6.70
$C_{3'''}/H_{3'''}$	146.0/-	146.0/-	144.3/-	146.2/-	146.4/-	146.0/-	146.1/-	145.64/-
$C_{4'''}/H_{4'''}$	142.0/-	144.6/-	142.8/-	144.7/-	144.9/-	144.5/-	144.7/-	144.18/-
$C_{5'''}/H_{5'''}$	117.1/6.68	116.5/6.68	116.7/6.63-7.05	116.4/6.68	116.7/6.68	116.3/6.67	116.3/6.67	116.56/6.68
$C_{6'''}/H_{6'''}$	121.2/6.56	121.3/6.57	121.6/6.63-7.05	121.3/6.56	121.6/6.57	121.3/6.53	121.3/6.57	121.50/6.57

$C_{7'''}/H_{7'''}$	72.3/4.05,3.70	72.2/3.71,4.04	71.6/-	72.3/3.71,4.08	72.6/4.00,3.72	71.8/4.04,3.64	72.4/4.00	72.32/3.75,3.96
$C_{8'''}/H_{8'''}$	36.5/2.79	36.5/2.80	35.0/2.73	36.6/2.79	36.9/2.79	36.3/2.70	36.6/2.79	36.12/2.79
$C_{1''''}/H_{1''''}$	111.4/5.22	112.1/5.11	109.4/5.80	111.4/5.21	111.4/4.90	111.0/4.90	111.0/4.90<br>112.0/5.15	111.80/5.09<br>110.55/4.90
$C_{2''''}/H_{2''''}$	78.5/3.65	77.8/3.93	77.3/-	78.6/3.68	78.4/3.87	78.1/3.87	78.1/3.73<br>78.1/3.96	77.93/3.93<br>77.83/3.86
$C_{3''''}/H_{3''''}$	79.9/3.65	80.5/3.73	79.9/-	80.2/-	80.9/-	80.6/-	80.6/-<br>80.2/-	80.56/-<br>80.56/-
$C_{4''''}/H_{4''''}$	74.8/3.39	75.0/3.95	74.1/-	74.8/3.60-3.65	75.4/3.92,3.74	75.1/3.93,3.74	75.1/3.92,3.75<br>74.9/4.05,3.92	74.75/3.72<br>74.75/3.72
$C_{5''''}/H_{5''''}$	65.7/3.27	65.6/3.57	64.3/-	65.8/3.31	66.0/3.54	65.7/3.55	65.6/3.54<br>65.2/3.59	65.13/3.57<br>65.13/3.54
Determination condition	75.5MHz, CD_3OD / 300.13MHz, CD_3OD	125MHz, CD_3OD / 500MHz, CD_3OD	100MHz, D_2O / 400MHz, D_2O	100MHz, D_2O / 400MHz, D_2O	125MHz, CD_3OD / 500MHz, CD_3OD	125 MHz, CD_3OD / 500 MHz, CD_3OD	75 MHz, CD_3OD / 500 MHz, CD_3OD	75MHz CD_3OD / 300MHz D_2O
Reference	[111]	[112]	[113]	[114]	[115]	[115]	[116]	[117]

Table S18. NMR Spectroscopic Data (δ) of Compounds 163~173

No.	163	164	165	166	167	168	169	170	171	172	173
Caffeoyl											
C_1/H_1	127.6/-	125.5/-	126.4/-	127.8/-	127.6/-	125.5/-	125.52/-	125.51/-	125.5/-	127.44/-	127.7/-
C_2/H_2	115.2/7.04	114.8/7.06	115.1/7.05	115.4/7.05	114.7/6.4-7.2	114.7/7.00	114.72/7.09	114.77/7.04	115.0/7.05	116.26/7.64	115.4/7.07
C_3/H_3	146.9/-	145.6/-	146.1/-	146.9/-	149.5/-	145.5/-	145.48/-	145.54/-	145.7/-	148.14/-	146.8/-
C_4/H_4	149.8/-	148.5/-	148.8/-	149.9/-	146.6/-	148.5/-	148.39/-	148.49/-	148.6/-	151.06/-	149.6/-
C_5/H_5	116.5/6.77	115.8/6.76	116.2/6.77	116.6/6.78	116.5/6.4-7.2	115.8/6.75	113.41/6.82	113.43/6.76	115.9/6.75	117.18/7.20	116.5/6.78
C_6/H_6	123.1/6.95	121.3/7.01	122.2/7.01	123.3/6.96	123.1/6.4-7.2	121.4/6.98	121.32/7.00	121.41/6.98	121.5/6.96	122.80/7.20	123.1/6.98
C_7/H_7	147.8/7.58	145.1/7.49	114.1/6.26	114.9/6.27	168.3/-	145.6/7.46	145.59/7.52	146.76/7.47	145.1/7.45	147.22/8.03	147.2/7.56
C_8/H_8	115.2/6.29	114.2/6.27	146.0/7.50	148.3/7.60	115.4/6.26	113.6/6.20	115.73/6.24	115.78/6.20	113.9/6.19	115.22/6.73	115.3/6.34
C_9/H_9	168.6/-	165.7/-	166.7/-	168.6/-	148.1/7.60	165.7/-	165.51/-	165.90/-	166.7/-	167.47/-	168.3/-
Glc											
$C_{1'}/H_{1'}$	104.4/4.35	100.1/4.74	102.7/4.39	104.3/4.39<br>104.8/4.30	104.1/4.28<br>104.5/4.38	101.3/4.57<br>101.8/4.54	102.37/4.41	102.24/4.35	102.7/4.28	104.76/4.81	103.5/4.49<br>104.3/4.63
$C_{2'}/H_{2'}$	75.2/3.27	73.3/4.65	76.6/3.08	76.3/3.39<br>75.2/3.20	75.9/-<br>74.9/-	81.1/3.56<br>74.3/2.99	74.36/3.28	74.42/3.23	74.0/3.12	76.13/4.02	82.6/3.67<br>75.4/3.09
$C_{3'}/H_{3'}$	75.8/3.60	74.1/3.42	73.5/3.66	81.7/3.81	81.5/-	77.7/3.86	78.88/3.77	78.95/3.72	81.1/3,40	80.84/4.47	81.3/4.09

				77.9/3.34	77.6/-	77.1/3.12					78.1/3.28
$C_{4'}/H_{4'}$	72.4/4.89	70.0/3.22	69.2/4.71	70.7/5.00<br>71.6/3.28	70.3/-<br>71.4/-	69.2/4.77<br>69.7/3.11	68.99/4.81	69.19/4.74	68.6/3.22	70.62/5.69	70.3/4.98<br>72.3/3.01
$C_{5'}/H_{5'}$	74.9/3.71	75.7/3.39	76.6/3.08	74.9/3.77<br>78.0/3.23	74.6/-<br>77.6/-	74.3/3.50<br>77.1/3.16	72.91/3.69	73.12/3.70	74.3/3.47	76.81/3.97	74.3/3.67<br>78.1/3.21
$C_{6'}/H_{6'}$	69.4/3.85,3.61	65.7/3.96,3.58	66.0/2.98,3.66	69.5/3.94,3.64<br>62.8/3.83,3.64	69.3/-<br>62.6/-	60.8/3.38,3.34<br>61.0/3.68,3.52	65.94/3.58-<br>3.39	67.95/3.65-<br>3.45	63.5/4.19,4.36	62.55/4.11,4.20	67.5/3.49,3.76<br>63.2/3.43,3.77
Xyl<br>$C_{1''}/H_{1''}$	105.3/4.24	104.0/4.20	104.2/4.15					103.82/4.12	105.9/4.26	108.01/5.18	104.9/4.53
$C_{2''}/H_{2''}$	74.9/3.15	73.3/2.98	73.6/2.96					73.22/2.97	71.3/3.37	73.76/4.51	73.2/3.56
$C_{3''}/H_{3''}$	77.6/3.30	76.6/3.09	76.7/3.68					76.31/3.08	72.7/3.33	75.23/4.10	74.1/3.47
$C_{4''}/H_{4''}$	71.1/3.45	69.6/3.28	69.7/3.25					69.48/3.23	67.8/3.59	70.17/4.23	69.8/3.73
$C_{5''}/H_{5''}$	66.9/3.14,3.82	68.2/3.70,3.02	68.3/3.45,3.66					65.62/2.98,3.64	65.8/3.36,3.65	67.84/3.72,4.25	67.4/3.16,3.77
Rhamnose<br>$C_{1'''}/H_{1'''}$				103.1/5.18	102.8/5.16	101.3/5.03	101.14/5.10<br>100.48/4.55	101.19/5.04	99.4/5.27	102.14/6.41	102.3/4.62
$C_{2'''}/H_{2'''}$				72.5/3.91	72.1/-	70.4/3.81	70.42/3.75<br>70.61/3.66	70.50/3.70	81.0/3.77	82.47/4.77	72.0/3.83

$C_{3'''}/H_{3'''}$				72.2/3.57	72.1/-	70.4/3.27	70.25/3.35<br>70.47/3.48	70.21/3.29	70.6/3.56	73.27/4.49	72.0/3.66
$C_{4'''}/H_{4'''}$				73.9/3.28	73.7/-	71.8/3.11	71.71/3.17<br>71.88/3.20	71.76/3.12	72.8/3.16	74.87/4.13	73.9/3.34
$C_{5'''}/H_{5'''}$				70.5/3.55	70.6/-	68.9/3.34	68.65/3.42<br>68.35/3.43	68.74/3.36	68.0/3.90	70.49/4.40	69.9/3.6
$C_{6'''}/H_{6'''}$				18.5/1.08	18.4/1.08	18.2/0.96	18.07/1.02<br>17.69/1.10	18.10/0.96	17.9/1.10	19.45/1.58	18.0/1.19
$C_{1''''}/H_{1''''}$	131.4/-	129.2/-	129.9/-	131.6/-	131.5/-	129.1/-	129.99/-	129.28/-	129.3/-	130.86/-	131.8/-
$C_{2''''}/H_{2''''}$	117.1/6.70	115.4/6.55	116.3/6.64	117.3/6.71	113.9/6.4-7.2	116.3/6.62	116.27/6.69	116.34/6.64	116.4/6.60	117.96/7.22	117.5/6.73
$C_{3''''}/H_{3''''}$	146.1/-	144.9/-	144.0/-	146.2/-	148.7/-	145.0/-	144.88/-	144.91/-	145.4/-	147.65/-	146.1/-
$C_{4''''}/H_{4''''}$	144.7/-	143.5/-	146.0/-	144.8/-	145.7/-	143.5/-	143.45/-	143.47/-	143.6/-	146.15/-	144.8/-
$C_{5''''}/H_{5''''}$	116.3/6.66	116.2/6.54	116.8/6.58	116.4/6.68	116.1/6.4-7.2	115.5/6.62	115.47/6.54	115.49/6.63	115.6/6.58	117.01/7.18	116.3/6.68
$C_{6''''}/H_{6''''}$	121.3/6.56	119.6/6.41	119.4/6.51	121.4/6.58	122.4/6.4-7.2	119.6/6.49	119.48/6.68	119.79/6.49	119.6/6.45	120.94/6.75	121.5/6.57
$C_{7''''}/H_{7''''}$	36.6/2.79	35.0/2.56	70.83/3.61,3.87	36.7/2.80	72.1/-	35.0/2.68	35.05/2.75	35.01/2.69	35.2/2.60	36.62/2.98	36.8/-
$C_{8''''}/H_{8''''}$	72.4/4.03,3.71	69.8/3.78,3.53	35.66/2.69	72.5/4.04,3.74	36.6/2.84	70.3/3.88,3.62	70.25/3.70,3.89	70.47/3.63-<br>3.88	70.4/3.58,3.78	71.81/3.91,4.30	72.3/3.7,4.01

$C_{9''''}/H_{9''''}$					56.6/3.84						
$C_{1'''''}/H_{1'''''}$			109.2/4.80<br>109.9/5.19								
$C_{2'''''}/H_{2'''''}$			73.6/2.96<br>74.4/3.24								
$C_{3'''''}/H_{3'''''}$			79.2/-<br>79.1/-								
$C_{4'''''}/H_{4'''''}$			73.8/3.56,3.78<br>74.0/3.56,3.78								
$C_{5'''''}/H_{5'''''}$			63.9/3.35<br>63.9/3.35								
Determination condition	100MHz, CD_3OD/ 400MHz, CD_3OD	100MHz, DMSO-d_6/ 400MHz, DMSO-d_6	100MHz, DMSO-d_6/ 500MHz, DMSO-d_6	MeOH-d_4/ 400MHz MeOH-d_4	22.5MHz Methanol-d_4/ 89.55MHz Methanol-d_4	100MHz DMSO-d_6/ 400MHz DMSO-d_6	90.53 MHz DMSO-d_6 +CF_3CO_2H/ 360 MHz DMSO-d_6 +CF_3CO_2H	90.53 MHz DMSO-d_6 +CF_3CO_2H/ 360 MHz DMSO-d_6 +CF_3CO_2H	150MHz, DMSO-d_6/ 600MHz, DMSO-d_6	150.35MHz Py-d_5 / 600MHz Py-d_5	125MHz, CD_3OD/ 500MHz, CD_3OD
Reference	[118]	[101]	[119]	[120]	[121]	[122]	[123]	[124]	[125]	[126]	[127]
